# Three-Year Overall Survival Outcomes and Correlative Analyses in Patients With NSCLC and High (50%–89%) Versus Very High (≥90%) Programmed Death-Ligand 1 Expression Treated With First-Line Pembrolizumab or Cemiplimab

**DOI:** 10.1016/j.jtocrr.2024.100675

**Published:** 2024-04-12

**Authors:** Biagio Ricciuti, Arielle Elkrief, Jessica Lin, Jianjun Zhang, Joao V. Alessi, Giuseppe Lamberti, Malini Gandhi, Alessandro Di Federico, Federica Pecci, Xinan Wang, Maisam Makarem, Cassio Murilo Hidalgo Filho, Teresa Gorria, Arushi Saini, Cindy Pabon, James Lindsay, Kathleen L. Pfaff, Emma L. Welsh, Mizuki Nishino, Lynette M. Sholl, Scott Rodig, Saadettin Kilickap, Petra Rietschel, Debra AG. McIntyre, Jean-Francois Pouliot, Mehmet Altan, Justin F. Gainor, John V. Heymach, Adam J. Schoenfeld, Mark M. Awad

**Affiliations:** a Lowe Center for Thoracic Oncology, Dana-Farber Cancer Institute, Boston, Massachusetts; b Thoracic Oncology Service, Department of Medicine, Memorial Sloan Kettering Cancer Center, New York, New York; c Department of Medicine, Center for Thoracic Cancers, Massachusetts General Hospital Cancer Center, Boston, Massachusetts; d Departments of Thoracic/Head & Neck Medical Oncology, The University of Texas MD Anderson Cancer Center, Houston, Texas; e Harvard T.H. Chan School of Public Health, Boston, Massachusetts; f Hospital Clinic de Barcelona, Barcelona, Spain; g ImmunoProfile, Department of Pathology, Brigham & Women’s Hospital and Dana-Farber Cancer Institute, Boston, Massachusetts; h Department of Radiology, Brigham and Women’s Hospital and Department of Imaging, Dana-Farber Cancer Institute, Boston, Massachusetts; i Department of Pathology, Brigham and Women’s Hospital, Boston, Massachusetts; j Faculty of Medicine, Department of Internal Medicine and Medical Oncology, Istinye University Istanbul, Istanbul, Turkey; k Regeneron Pharmaceuticals, Tarrytown, New York

**Keywords:** PD-L1, Cemiplimab, Pembrolizumab, Long-term outcomes, Biomarkers

## Abstract

**Introduction:**

Responses to first-line programmed cell death protein 1 inhibition vary among patients with metastatic NSCLC and a programmed death-ligand 1 (PD-L1) tumor proportion score (TPS) greater than or equal to 50%. We previously reported improved clinical outcomes to first-line programmed cell death protein 1 inhibition in patients with metastatic NSCLC with a PD-L1 TPS of greater than or equal to 90% versus 50% to 89% in a pilot study. Here, we report the three-year survival with first-line pembrolizumab and cemiplimab in two large independent cohorts of patients with PD-L1 TPS greater than or equal to 90% versus 50% to 89% and characterize genomic and immunophenotypic differences between these PD-L1 expression groups, which were largely unknown.

**Methods:**

We analyzed three-year outcomes of the following two independent cohorts: (1) a multicenter cohort of patients from four academic centers in the United States treated with pembrolizumab and (2) EMPOWER-Lung 1, randomized, phase III trial comparing first-line cemiplimab with chemotherapy. Tumor genomic profiling and multiplexed immunofluorescence were performed to evaluate genomic and immunophenotypic correlates of very high PD-L1 expression.

**Results:**

At three years of follow-up, progression-free survival (hazard ratio [HR], 0.69; *p* < 0.001) and overall survival (HR, 0.70; *p* < 0.01) to first-line commercial pembrolizumab were significantly improved in patients with a PD-L1 TPS greater than or equal to 90% versus 50% to 89%. In the EMPOWER-Lung 1, patients assigned to the cemiplimab arm with a PD-L1 TPS greater than or equal to 90% also had significant improvements in progression-free survival (HR, 0.53; *p* < 0.0001) and overall survival (HR, 0.63; *p* = 0.007) compared with those with a PD-L1 of 50% to 89%. Tumor genomic profiling of 553 NSCLC samples revealed that mutations in *STK11* and *SMARCA4* were significantly more frequent in tumors with a PD-L1 TPS of 50% to 89% compared with those with a PD-L1 TPS greater than or equal to 90% (Q < 0.15), whereas *BRCA2* was enriched in NSCLC samples with a PD-L1 TPS greater than or equal to 90% (Q < 0.15). Multiplexed immunofluorescence on 93 NSCLC samples identified higher intratumoral CD8^+^PD1^+^ T cells (*p* = 0.02) in tumors with PD-L1 TPS greater than or equal to 90% versus 50% to 89%.

**Conclusion:**

Pembrolizumab and cemiplimab were found to have long-term survival benefit and favorable genomic and immunophenotypic profile in patients with advanced NSCLC with PD-L1 TPS greater than or equal to 90% compared with TPS 50% to 89%.

## Introduction

The introduction of programmed cell death protein 1 (PD-1) and programmed death-ligand 1 (PD-L1) monoclonal antibodies either alone or in combination with chemotherapy has changed the first-line treatment landscape of patients with advanced NSCLC. For patients whose tumors have a high PD-L1 tumor proportion score (TPS) of greater than or equal to 50%, PD-(L)1 monotherapy represents one of the approved options which has the advantage of sparing the side effects of platinum doublet chemotherapy.^1^, ^2^, ^3^, ^4^ For patients with negative (<1%) or low (1%–49%) PD-L1 expression, a combination of platinum doublet chemotherapy and a PD-(L)1 inhibitor is generally favored, given that PD-(L)1 monotherapy may not be as effective in this patient population.^2^^,^^5^, ^6^, ^7^ Unfortunately, even among patients with a high PD-L1 TPS greater than or equal to 50%, responses occur in only approximately 45% of cases.^1^

Our group has previously revealed that among patients with advanced NSCLC with PD-L1 TPS greater than or equal to 50%, cases with a very high PD-L1 TPS greater than or equal to 90% have significantly higher objective response rate (ORR) and longer median progression-free survival (PFS) and overall survival (OS) compared with those with PD-L1 TPS of 50% to 89%.^8^ Similarly, in the more recent randomized, phase III EMPOWER-Lung 1 trial, which evaluated the PD-1 inhibitor cemiplimab versus platinum-based chemotherapy in untreated, advanced NSCLC with PD-L1 expression greater than or equal to 50%, increasing PD-L1 expression levels (50%–60%, 61%–89%, and ≥90%) were found to correlate with incremental improvements in ORR, PFS, and OS.^4^ Thus far, no systematic analysis has been performed to compare the long-term benefit from PD-1 inhibition among patients with high (50%–89%) versus very high (≥90%) PD-L1 TPS. In addition, whether tumors with very high PD-L1 TPS greater than or equal to 90% have unique genomic and immunophenotypic characteristics compared with those with a PD-L1 TPS of 50% to 89% is also unknown.

Here, we report the three-year survival outcomes of patients with advanced NSCLC who had a PD-L1 TPS of 50% to 89% vs greater than or equal to 90% and received first-line PD-1 inhibition in two independent cohorts, including a multi-institutional, retrospective cohort of patients treated with commercial first-line pembrolizumab and the prospective, randomized, phase III EMPOWER-Lung 1 trial of cemiplimab versus chemotherapy. In addition, we performed clinicogenomic and immunophenotypic characterization of NSCLC with PD-L1 TPS greater than or equal to 50% to identify features that are unique to NSCLC with a PD-L1 TPS greater than or equal to 90% versus 50% to 89%.

## Methods

### Patient Population

#### Retrospective Academic Cohort

Clinicogenomic and immunophenotypic data were collected from patients with NSCLC who had consented to correlative research studies at the Dana-Farber Cancer Institute (DFCI), Memorial Sloan Kettering Cancer Center (MSKCC), MD Anderson Cancer Center (MDACC), and Massachusetts General Hospital (MGH). Eligible patients were aged 18 years or older, had histologically or cytologically confirmed stage IV squamous or nonsquamous NSCLC without *EGFR* mutation or *ALK* fusions (as per KEYNOTE 024) with PD-L1 expressed in at least 50% of tumor cells, and had received first-line pembrolizumab monotherapy. All subjects gave their informed consent for inclusion before they participated in the study. The study was conducted in accordance with the Declaration of Helsinki.

#### EMPOWER-Lung 1

EMPOWER-Lung 1 is a multicenter, open-label, global, phase III study. Patients recruited in 138 centers from 24 countries were randomly assigned (1:1) to cemiplimab or platinum-doublet chemotherapy as first-line treatment for NSCLC.

Eligible patients were aged 18 years or older, had histologically or cytologically confirmed advanced squamous or nonsquamous NSCLC with PD-L1 expressed in at least 50% of tumor cells, and an Eastern Cooperative Oncology Group (ECOG) performance status (PS) score of 0 or 1. Patients were ineligible if they had never smoked, had active untreated brain metastases, or had tumors which were positive for *EGFR* mutations or *ALK/ROS1* translocations. All subjects gave their informed consent for inclusion before they participated in the EMPOWER-Lung 1 study. Details of eligibility criteria and study design are published elsewhere.^4^

### PD-L1 Tumor Proportion Score Assessment and Cutoff Selection

In the academic cohort, the PD-L1 TPS by immunohistochemistry (IHC) was scored by trained pathologists using validated monoclonal anti–PD-L1 antibodies: E1L3N (Cell Signaling Technology, Danvers, MA), 22C3 (Dako North America Inc., Carpinteria, CA), and 28-8 (Epitomics Inc., Burlingame, CA). In EMPOWER-Lung 1, the PD-L1 TPS by IHC was scored by trained pathologists using the 22C3 antibody.

The PD-L1 cutoff of 90% was previously established by our group using unbiased recursive partitioning algorithm^8^ and independently validated in the prospective EMPOWER-Lung 01 trial, as previously revealed.^4^

### Tumor Genomic Profiling and Tumor Mutational Burden Assessment

Comprehensive targeted exome next-generation sequencing was performed on a separate cohort of patients with matched PD-L1 IHC using the validated OncoPanel assay at the DFCI, as previously described.^9^ Tumor mutational burden (TMB), defined as the number of somatic mutations per megabase (Mb) of genome examined (including coding, base substitution, and indels), was determined using OncoPanel (DFCI cohort) and MSK-IMPACT (MSKCC cohort).^9^^,^^10^

Copy number variants and structural variants were called using the internally developed algorithms RobustCNV and BreaKmer. For each gene, the absolute copy number (ACN) was estimated based on the tumor purity (p) and the weighted average of segmented log2 ratios across the gene (l) using the following formula:

$$ACN\;=\;\frac{2^{\left(I\;+\;1\right)}-2\left(1-p\right)}{p}$$

To quantify aneuploidy levels and determine aneuploidy score, sequencing data were analyzed using Arm-level Somatic Copy-number Events in Targeted Sequencing (ASCETS), as previously described.^11^

### Multiplexed Immunofluorescence (ImmunoProfile)

Multiplexed immunofluorescence (mIF) was performed on samples from a separate cohort of NSCLC at DFCI by staining 5-micron formalin-fixed, paraffin-embedded (FFPE) whole tissue sections with nuclear counterstain/4′,6-diamidino-2-phenylindole (DAPI), PD-L1 (clone E1L3N), PD-1 (clone EPR4877[2]), CD8 (clone 4B11), FOXP3 (clone D608R), and Cytokeratin (clone AE1/AE3). Regions of Interest (ROIs) were defined for each image. A custom script quantified the number/percentage of positive cells for relevant biomarkers in specific tissue regions. Each ROI was divided into one or more of these defined regions: intratumoral (IT), defined as the region of the slide consisting of tumor beyond the tumor-stroma interface; tumor-stroma interface (TSI), defined as the region within 40 microns to either side of the defined border between tumor and stroma; and total (IT + TSI). Cell count was calculated per ROI and averaged across ROIs, reported as count per millimeter squared ± SE.

### Statistical Analysis

Categorical and continuous variables were summarized using descriptive statistics. The Wilcoxon test and the Kruskal-Wallis test were used to test for differences between continuous variables, and Fisher’s exact test was used to test for associations between categorical variables. PFS was defined as the time between the date of treatment initiation and the date of disease progression or death, whichever occurred first. Patients without disease progression were censored at the time of their last disease assessment. OS was defined as the time between treatment initiation and death or last contact. The Kaplan-Meier methodology was used to estimate event-time distributions. Log-rank tests were used to test for differences in event-time distributions, and Cox proportional-hazards models were fitted to obtain estimates of hazard ratios (HRs) in univariate and multivariable models. Mutation enrichment analysis was performed using the R package maftools^12^ and was restricted to nonsquamous NSCLC. Statistical significance was defined as *p* less than 0.05. Propensity score matching was performed using the R package MatchIt. All statistical analyses were performed using R version 3.6.1

## Results

### Patient Characteristics

A total of 516 patients with advanced NSCLC and a PD-L1 TPS greater than or equal to 50% who received first-line pembrolizumab monotherapy at four academic centers in the United States and had a minimum follow-up of 36 months were included in the retrospective cohort. Among these, the median age was 69 years, 52.5% were female, 91.7% had a history of tobacco use, 80.6% had an ECOG PS of 0 to 1, and 85.3% had nonsquamous histology. A PD-L1 TPS of 50% to 89% was observed in 61.8%, whereas 38.2% of the cases had a PD-L1 TPS greater than or equal to 90%. Baseline clinicopathologic features of these patients are summarized in Supplementary Table 1.

**Figure 1:**
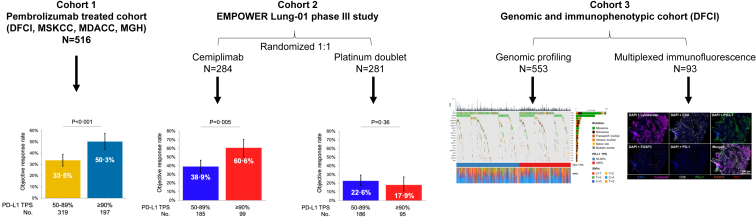
Study schema. Patients with metastatic NSCLC and a PD-L1 TPS greater than or equal to 50% who were treated with first-line pembrolizumab at DFCI, MSKCC, MDACC, and MGH were included in cohort 1. Patients who enrolled in the randomized, phase III trial EMPOWER-Lung 01 of first-line cemiplimab versus chemotherapy were included in cohort 2 (independent validation cohort). Cohort 3 included a separate subset of patients with NSCLC who underwent full tumor genomic profiling and multiplexed immunofluorescence at DFCI and had matched PD-L1 expression assessment. This cohort was used to determine the genomic and immunophenotypic correlates of very high and high PD-L1 TPS. DFCI, Dana-Farber Cancer Institute; MDACC, MD Anderson Cancer Center; MGH, Massachusetts General Hospital; MSKCC, Memorial Sloan Kettering Cancer Center; PD-L1, programmed death-ligand 1; TPS, tumor proportion score.

A total of 565 patients with a PD-L1 expression greater than or equal to 50% were enrolled in the EMPOWER-Lung 1 study, of whom 284 (50.3%) were randomized to receive single-agent cemiplimab and 281 (49.7%) to receive platinum-based chemotherapy. In the cemiplimab arm, the median age was 63 years, 12.3% were women, 56.7% had nonsquamous histology, and 34.9% had a PD-L1 TPS greater than or equal to 90%. In the chemotherapy arm, the median age was 64 years, 17.8% were women, 56.6% had nonsquamous histology, and 33.8% had a PD-L1 TPS greater than or equal to 90%. In both arms, all patients had a history of tobacco use (Supplementary Table 2). A study schema is found in Figure 1.

### Three-Year Survival Outcomes to First-Line Pembrolizumab Monotherapy in NSCLC With PD-L1 TPS 50% to 89% Versus Greater Than or Equal to 90%

We first explored the impact of increasing PD-L1 expression levels on three-year survival outcomes among patients with NSCLC treated with commercial pembrolizumab monotherapy. Baseline characteristics including age, sex, histology, performance status, TMB, and driver gene alterations were well balanced between cases with a PD-L1 TPS 50% to 89% and greater than or equal to 90% (Supplementary Table 3, Supplementary Fig. 1). Nevertheless, patients with a PD-L1 TPS greater than or equal to 90% were more likely to be ever smokers compared with those with a PD-L1 TPS of 50% to 89% (94.9% versus 89.7%; *p* = 0.047).

**Figure 2:**
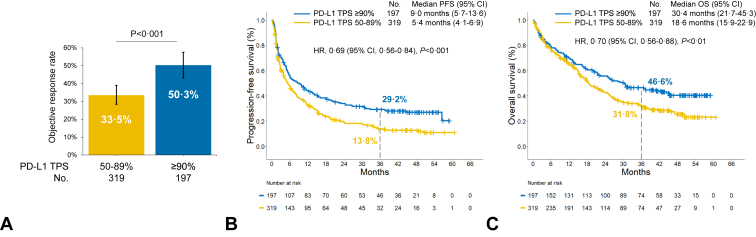
(*A*) Objective response rate, (*B*) three-year progression-free survival rates, and (*C*) three-year overall survival rates to first-line pembrolizumab in patients with advanced NSCLC and PD-L1 TPS of greater than or equal to 90% versus 50% to 89% in the retrospective cohort from academic sites. CI, confidence interval; HR, hazard ratio; OS, overall survival; PD-L1, programmed death-ligand 1; TPS, tumor proportion score.

In this first-line pembrolizumab cohort, with a minimum follow-up of three years (median follow-up 43.7 mo), the ORR was significantly higher among patients with very high PD-L1 expression of greater than or equal to 90% compared with those with PD-L1 TPS of 50% to 89% (50.3% versus 33.5%; *p* < 0.001, Fig. 2*A*). A very high PD-L1 TPS was associated with a significantly longer median PFS (9.0 versus 5.4 mo; HR, 0.69; *p* < 0.001) and median OS (30.4 versus 18.6 mo; HR, 0.70; *p* < 0.01) compared with a PD-L1 TPS of 50% to 89% (Fig. 2*B* and *C*). Kaplan-Meier estimates of PFS at three years were 29.2% (95% confidence interval [CI], 23.3%–36.6%) in the PD-L1 TPS greater than or equal to 90% group and 13.8% (95% CI, 10.3%–18.6%) in the PD-L1 TPS 50% to 89% group, whereas OS estimates at three years were 46.6% (95% CI, 39.9%–54.3%) and 31.8% (95% CI, 26.7%–37.8%), respectively, in these two groups. Patients in the PD-L1 TPS greater than or equal to 90% group were significantly more likely to have completed two years of pembrolizumab monotherapy compared with those in the PD-L1 TPS 50% to 89% group (20.3% versus 10.6%; *p* = 0.004, Supplementary Fig. 2).

The survival benefit of the pembrolizumab monotherapy in patients with a PD-L1 TPS greater than or equal to 90%, compared with 50% to 89%, was observed in most key subgroups that were analyzed, including age, sex, and *KRAS* mutation status, and among patients with a history of tobacco use, nonsquamous histology, and an ECOG PS of 0 to 1. In never smokers, and in patients with an ECOG PS of greater than or equal to 2 or squamous histology, there was no significant difference in PFS or OS between the PD-L1 TPS 50% to 89% versus greater than or equal to 90% groups (Supplementary Fig. 3*A* and *B*), though the small sample size of these subgroups may have affected these results.

Because the pembrolizumab outcomes were collected from retrospective data, multivariable Cox regression analysis for PFS and OS was performed to adjust for potential confounders in this nonrandomized cohort. After adjusting for confounders, very high PD-L1 TPS greater than or equal to 90% retained a significant association with improved PFS (HR, 0.70; *p* = 0.001) and OS (HR, 0.73; *p* = 0.01) in multivariable analysis (Supplementary Table 4). For additional sensitivity testing, we also conducted a 1:1 propensity score matching analysis to ensure appropriate balance between treatment groups and found that a very high PD-L1 TPS greater than or equal to 90% was associated with a significant improvement in ORR (50.3% versus 29.4%; *p* < 0.001), PFS (9.0 versus 5.0 mo; HR, 0.71; *p* < 0.01), and OS (30.4 versus 19.0 mo; HR, 0.73; *p* = 0.018) with first-line pembrolizumab at three years of follow-up compared with a PD-L1 TPS of 50% to 89% (Supplementary Fig. 4). Furthermore, in these propensity-matched cohorts, a PD-L1 TPS greater than or equal to 90% was an independent predictor of immunotherapy efficacy in multivariable analysis (Supplementary Table 5).

### Three-Year Survival Outcomes to First-Line Cemiplimab Monotherapy in NSCLC With PD-L1 TPS 50% to 89% Versus Greater Than or Equal to 90% in EMPOWER-Lung 1

**Figure 3:**
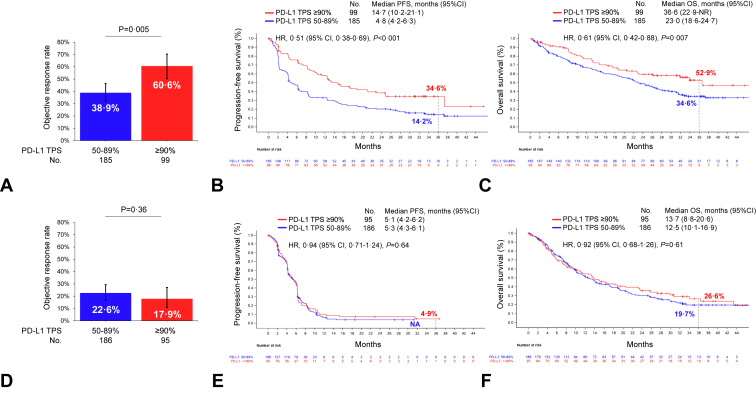
(*A*) Objective response rate, (*B*) three-year progression-free survival rates, and (*C*) three-year overall survival rates to first-line cemiplimab in patients with advanced NSCLC and PD-L1 TPS of greater than or equal to 90% versus 50% to 89% in the EMPOWER-Lung 1 study. (*D*) Objective response rate, (*E*) three-year progression-free survival rates, and (*F*) three-year overall survival rates to first-line chemotherapy in patients with advanced NSCLC and PD-L1 TPS of greater than or equal to 90% versus 50% to 89% in the EMPOWER-Lung 1 study. CI, confidence interval; HR, hazard ratio; OS, overall survival; PD-L1, programmed death-ligand 1; TPS, tumor proportion score.

To confirm these findings, we next explored three-year survival patients who received first-line cemiplimab in the randomized, phase III study EMPOWER-Lung 1 study, comparing outcomes in the PD-L1 TPS 50% to 89% versus greater than or equal to 90% groups. Baseline clinicopathologic characteristics were well balanced between these groups (Supplementary Table 6). At the three-year follow-up (median follow-up 37 mo), the ORR was significantly higher among patients with a very high PD-L1 TPS greater than or equal to 90% compared with 50% to 89% (60.6% versus 38.9%; *p* = 0.005, Fig. 3*A*). A very high PD-L1 TPS greater than or equal to 90% was also associated with a significantly longer mPFS (14.7 versus 4.8 mo; HR, 0.51; *p* < 0.001) and mOS (36.6 versus 23.0 mo; HR, 0.61; *p* = 0.007) compared with a PD-L1 TPS of 50% to 89% (Fig. 3*B* and *C*). Kaplan-Meier estimates of PFS at three years were 34.6% (95% CI, 24.8–44.6) in the PD-L1 TPS greater than or equal to 90% group and 14.2% (95% CI, 3.3–20.1) in the PD-L1 TPS 50% to 89% group, whereas OS estimates at three years were 52.9% (95% CI, 40.4–64.0) and 34.6% (95% CI, 27.1–42.1), respectively, in these two groups. The benefit of cemiplimab monotherapy in patients with a PD-L1 TPS greater than or equal to 90% was observed across most key subgroups that were analyzed, except females (Supplementary Fig. 5*A* and *B*), though this may simply reflect the very small proportion of women enrolled in the EMPOWER-Lung 01 (approximately 12%). The proportion of patients who completed two years of cemiplimab treatment was not significantly different among patients with a PD-L1 TPS greater than or equal to 90% versus 50% to 89% (29.3% versus 20.5%; *p* = 0.09, Supplementary Fig. 6).

Importantly, we did not observe differences in ORR (22.6% versus 17.9%; *p* = 0.36), mPFS (HR, 0.94; *p* = 0.64), or mOS (HR, 0.92; *p* = 0.61) at three years of follow-up in the PD-L1 TPS 50% to 89% versus greater than or equal to 90% groups among patients who received platinum-based chemotherapy in the control arm of EMPOWER-Lung 1, suggesting that very high PD-L1 TPS greater than or equal to 90% is predictive of long-term benefit from cemiplimab monotherapy, and not prognostic (Fig. 3*D*–*F*). No meaningful benefit was observed for any key subgroup in the chemotherapy arm according to PD-L1 expression level (Supplementary Fig. 7). Baseline clinical characteristics of patients treated with chemotherapy according to PD-L1 expression (50%–89% versus greater than or equal to 90%) are summarized in Supplementary Table 7. Of note, patients with PD-L1 greater than or equal to 90% in the chemotherapy arm had numerically lower ORR and experienced more early deaths. At approximately 10 months, the survival curves inversed which is approximately the median time to crossover from chemotherapy to cemiplimab in the EMPOWER-LUNG-1 study. The long-term survival that favors the PD-L1 greater than or equal to 90% subgroup was likely due to the impact of second-line cemiplimab.

### Outcomes to First-Line Immunotherapy PD-L1 TPS 50% to 60%, 61% to 89%, and Greater Than or Equal to 90% Groups

We next asked whether progressively increasing PD-L1 TPS expression levels of 50% to 60%, 61% to 89%, and greater than or equal to 90%, as previously reported,^4^ were associated with progressively improved outcomes at longer follow-up. Among patients treated with first-line pembrolizumab, there was a progressive improvement in ORR, PFS, and OS with increasing PD-L1 expression categories, with those in the very high PD-L1 TPS greater than or equal to 90% group having the best long-term outcomes (Supplementary Fig. 8*A*–*C*). Similar results were observed in the EMPOWER-Lung 1 study according to gradually increasing PD-L1 levels (Supplementary Fig. 9*A*–*C*). By contrast, there was no difference in ORR or three-year PFS or OS in the chemotherapy arm by increasing PD-L1 expression levels in EMPOWER-Lung 1 (Supplementary Fig. 9*D*–*F*).

### Genomic Profiles of NSCLCs With a PD-L1 TPS of 50% to 89% Versus Greater Than or Equal to 90%

**Figure 4:**
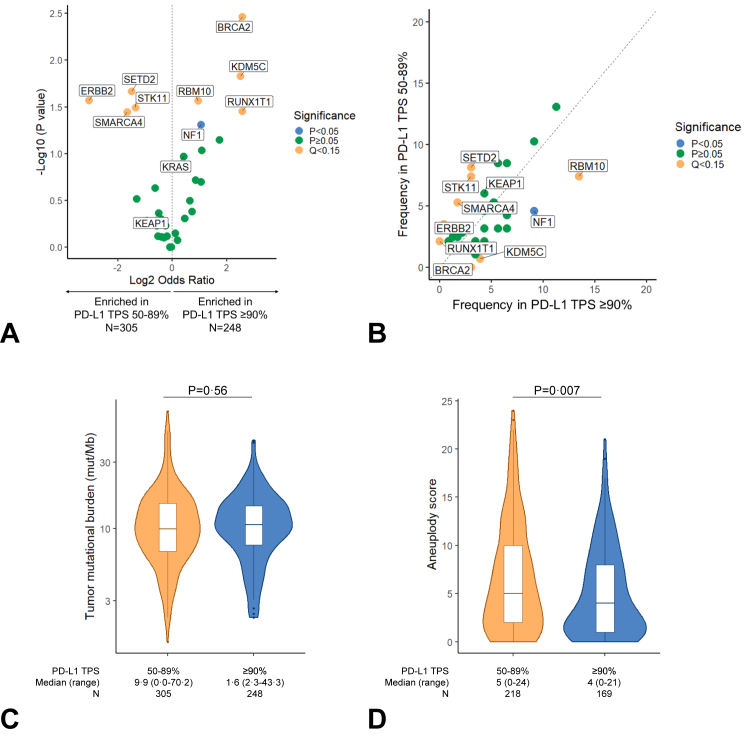
(*A*) Volcano plot illustrating gene mutations that are enriched in nonsquamous NSCLC with a PD-L1 TPS of greater than or equal to 90% versus 50% to 89%. (*B*) Frequency of gene mutations enriched in nonsquamous NSCLC with a PD-L1 TPS of greater than or equal to 90% versus 50% to 89%. (*C*) Tumor mutational burden and (*D*) aneuploidy score distributions in NSCLC samples with a PD-L1 TPS of 50% to 89% versus greater than or equal to 90%. PD-L1, programmed death-ligand 1; TPS, tumor proportion score.

We next explored whether NSCLCs with PD-L1 expression greater than or equal to 90% had different genomic profiles compared with those with a PD-L1 expression level of 50% to 89%. A separate cohort of 553 NSCLC samples with matched tumor genomic profiling and PD-L1 expression on the same tissue at the DFCI was included in this analysis; their clinicopathologic characteristics are summarized in Supplementary Table 8. These groups were well balanced in terms of baseline characteristics. As expected, there were fewer never smokers among patients with a PD-L1 TPS greater than or equal to 90% versus 50% to 89% (13.3% versus 20.3%, *p* = 0.03). In this cohort, the most common mutations included *TP53* (67%), *KRAS* (42%), *EGFR* (12%), *CDKN2A* (10%), *RBM10* (10%), and *ARID1A* (7%) (Supplementary Fig. 10). Gene mutations that were significantly enriched in the PD-L1 TPS of 50% to 89% group of nonsquamous NSCLCs included *ERBB2*, *STK11*, *SMARCA4,* and *SETD2* (Q < 0.15, Fig. 4*A* and *B*). By contrast, mutations in *BRCA2*, *RBM10*, *KDM5C*, and *RUNX1T1* were enriched among samples with a PD-L1 TPS greater than or equal to 90% (Q < 0.15, Fig. 4*A* and *B*). *NF1* mutations were enriched among cases with a PD-L1 TPS greater than or equal to 90% (*p* < 0.05), although this was not significant after false discovery rate (FDR) adjustment. In this genomic cohort of samples, there was no significant difference in TMB between samples with a PD-L1 TPS of 50% to 89% versus greater than or equal to 90% (Fig. 4*C*). Nevertheless, among cases assessable for aneuploidy levels (N = 387), there was a significantly lower aneuploidy score among patients with a very high PD-L1 TPS greater than or equal to 90% (Fig. 4*D*).

### Immunophenotypic Characteristics of NSCLCs With a PD-L1 TPS of 50% to 89% Versus Greater Than or Equal to 90%

**Figure 5:**
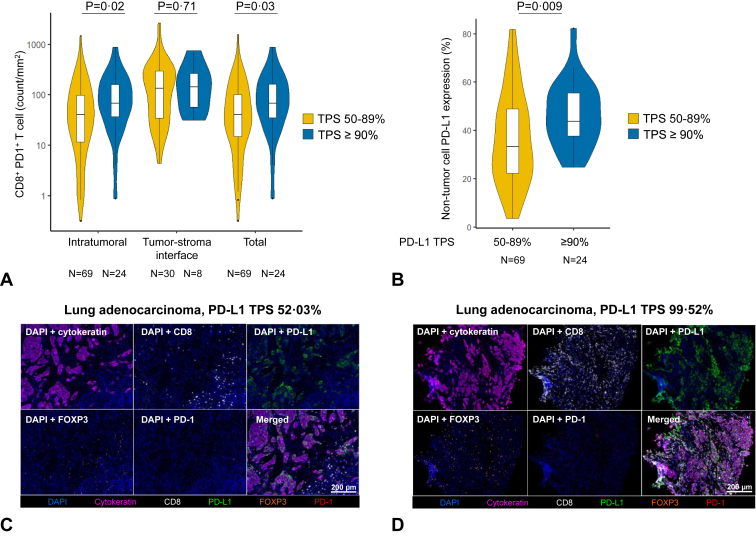
(*A*) Intratumoral, tumor-stroma interface, and total CD8^+^PD1^+^ T cells in NSCLC samples with a PD-L1 TPS greater than or equal to 90% versus 50% to 89%. (*B*) Proportion of PD-L1^+^ non-tumor cells in NSCLC samples with a PD-L1 TPS greater than or equal to 90% versus 50% to 89%. Representative cases of lung adenocarcinoma samples with (*C*) PD-L1 TPS greater than or equal to 90% or (*D*) 50% to 89% which underwent multiplexed immunofluorescence. PD-L1, programmed death-ligand 1; TPS, tumor proportion score.

We last investigated whether NSCLCs with a PD-L1 TPS of greater than or equal to 90% had distinct immunophenotypic correlates compared with those with a PD-L1 TPS of 50% to 89% and performed mIF for CD8, PD-1, Foxp3, PD-L1, and AE1/AE3 (Cytokeratin) on tumor tissue from 69 NSCLCs with PD-L1 TPS of 50% to 89% and 24 NSCLCs with PD-L1 TPS greater than or equal to 90%. The clinicopathologic characteristics of these 93 patients are presented in Supplementary Table 9. Compared with NSCLCs with PD-L1 TPS of 50% to 89%, NSCLCs with PD-L1 expression greater than or equal to 90% had significantly greater intratumoral CD8^+^PD-1^+^ T cells (median 67.6 versus 30.7 cells/mm^2^, *p* = 0.02) and total CD8^+^PD-1^+^ cells (67.6 versus 31 cells/mm^2^; *p* = 0.03) (Fig. 5*A*). There was no significant difference in intratumoral, tumor-stroma interface, and total PD-1^+^ cells, Foxp3+, and CD8^+^ T cells between the two groups, though these were numerically higher among tumors with PD-L1 TPS greater than or equal to 90% (Supplementary Fig. 11*A*–*C*). Median PD-L1 expression on non-tumor cells was significantly higher in cases with PD-L1 TPS of greater than or equal to 90% compared with those with PD-L1 TPS 50% to 89% (43.7% versus 33.4%; *p* = 0.009, Fig. 5*B*). On a continuous scale, there was also a positive linear correlation between PD-L1 TPS and immune cell subsets in all comers with NSCLC (Supplementary Figs. 12 and 13). Representative mIF images of lung adenocarcinoma cases with PD-L1 TPS of 50% to 89% and TPS greater than or equal to 90% are found in Figure 5*C* and *D*, respectively.

In conclusion, this analysis reveals that patients with advanced NSCLC and a very high PD-L1 TPS of greater than or equal to 90% have significantly improved OS at three years of follow-up from first-line pembrolizumab or cemiplimab monotherapy compared with those with a PD-L1 TPS of 50% to 89%. The three-year OS rates among patients with a PD-L1 greater than or equal to 90% were 46.6% with pembrolizumab and 52.9% with cemiplimab and only 26.6% in patients receiving first-line platinum doublet chemotherapy in the control arm of the EMPOWER-Lung 1 despite 74% crossover. Impressively, the three-year survival rates for patients with a PD-L1 greater than or equal to 90% treated with PD-1 monotherapy are close to the three-year OS rates of patients with *EGFR-*mutant or *ALK*-positive NSCLC receiving osimertinib or the first-generation ALK inhibitor crizotinib, respectively, which range from 50% to 60%.^13^^,^^14^

In this study, we also found that the proportion of patients who completed two years of treatment with first-line pembrolizumab was higher in the PD-L1 TPS greater than or equal to 90% group compared with the PD-L1 TPS 50% to 89% group in two independent cohorts (20% versus 10% in the real world, retrospective academic cohort, and 30% versus 20% in the cemiplimab arm of the phase III EMPOWER-Lung 1). In comparison, in the recent five-year updates of the KEYNOTE-189 and -407 studies comparing chemo-immunotherapy with standard chemotherapy among patients with nonsquamous and squamous NSCLC, the proportion of patients completing 2 years of treatment in the PD-L1 greater than or equal to 50% was 23.4% and 21.9%, respectively.^15^^,^^16^ In the absence of direct head-to-head comparisons between PD-(L)1 inhibitor monotherapy and chemo-immunotherapy in patients with advanced NSCLC and a PD-L1 TPS greater than or equal to 50% or greater than or equal to 90%, our data indicate that PD-1 inhibitor monotherapy with cemiplimab or pembrolizumab will continue to represent an appropriate safe and effective therapeutic standard option in patients with very high PD-L1 expression.

Our results also have implications for clinical trial design and interpretation. There are several prospective studies evaluating the safety and efficacy of PD-(L)1 inhibitors alone or in combination with novel agents in patients with advanced or metastatic NSCLC. Our findings suggest that very high PD-L1 expression greater than or equal to 90% should be considered as a stratification factor in these studies, especially when comparing monotherapies with novel combination strategies; stratifying according to less granular PD-L1 expression subgroups, such as TPS less than 1%, 1% to 49%, and greater than or equal to 50% may not ensure an accurate balance between treatment arms. Importantly, in this study, we observed similar outcomes between patients with a PD-L1 of 61% to 89% and those with a PD-L1 of 50% to 60% suggesting the benefit from PD-1 monotherapy is driven by the very high expressors with a TPS greater than or equal to 90%. This indicates that other thresholds such as greater than or equal to 75% or greater than or equal to 80% may not necessarily capture the true benefit from first-line PD-1 inhibition.

This study also has important ramifications for the management of patients with early stage NSCLC. Several randomized phase III trials of adjuvant and neoadjuvant immunotherapy have revealed how PD-(L)1 blockade alone or in combination with chemotherapy leads to significant substantial in disease-free survival and OS in patients with stages IB to IIIA NSCLC.^17^, ^18^, ^19^ Nevertheless, robust and clinically available biomarkers are not currently available to help distinguish patients who derive the greatest benefit from (neo)adjuvant immune checkpoint blockade versus those who may not need perioperative immunotherapies. Subgroup analyses from these studies indicate that a PD-L1 TPS greater than or equal to 50% may potentially identify patients with the lowest risk of recurrence after both adjuvant and neoadjuvant PD-(L)1 inhibition.^17^^,^^19^ In this context, our results suggest that progressively increasing PD-L1 thresholds should be explored as predictors of immunotherapy efficacy also among patients with early stage lung cancer, to inform the design of perioperative trials with immune checkpoint blockade.

In this study, we also noted that NSCLCs with very high PD-L1 levels greater than or equal to 90% are enriched in *BRCA2* and *KDM5C* loss-of-function mutations, which have been previously associated with increased mutational burden, increased CD8+ T cell infiltration, effector T-cell signatures, and improved outcomes to immunotherapy in NSCLC.^20^, ^21^, ^22^, ^23^ By contrast, NSCLCs with a PD-L1 TPS of 50% to 89% were enriched in loss-of-function mutations in *STK11* and *SMARCA4* and have higher aneuploidy levels, which are mediators of primary resistance to PD-(L)1 blockade in NSCLC.^24^^,^^25^ We also found that NSCLC samples with a very high PD-L1 TPS had increased levels of intratumoral CD8^+^PD1^+^ T cells and PD-L1^+^ non-tumor cells. These data highlight how NSCLCs with very high PD-L1 expression have unique genomic and immunophenotypic features that contribute to the significantly longer survival with immunotherapies observed in these patients.

Limitations of this study include the retrospective design and the relatively small size of samples that underwent immunophenotypic characterization. In addition, different PD-L1 clones were used for PD-L1 assessment depending on Institutional practices. Nevertheless, there is strong analytical evidence for interchangeability for the clones that were used in this study. Last, the immunophenotypic characterization was performed on archival samples from patients not treated with immunotherapy, which limited the possibility to perform additional correlative analysis.

In conclusion, in this report, we reveal that among patients with metastatic NSCLC treated with first-line PD-1 inhibition, a PD-L1 TPS greater than or equal to 90% is associated with a clinically meaningful survival benefit at three years of follow-up and more favorable immunologic profiles. These results can help guide treatment decisions and inform trial interpretation and design.

## CRediT Authorship Contribution Statement

**Biagio Ricciuti:** Conceptualization, Methodology, Project administration, Supervision, Visualization, Writing—original draft, Writing—review and editing.

**Arielle Elkrief:** Conceptualization, Methodology, Project administration, Supervision, Visualization, Writing—original draft, Writing—review and editing.

**Jessica Lin:** Conceptualization, Methodology, Project administration, Supervision, Visualization, Writing—original draft, Writing—review and editing.

**Jianjun Zhang:** Supervision, Data collection, Visualization, Writing—original draft, Writing—review and editing.

**Joao V. Alessi:** Supervision, Data collection, Visualization, Writing—original draft, Writing—review and editing.

**Giuseppe Lamberti:** Supervision, Data collection, Visualization, Writing—original draft, Writing—review and editing.

**Malini Gandhi:** Supervision, Data collection, Visualization, Writing—original draft, Writing—review and editing.

**Alessandro Di Federico:** Supervision, Data collection, Visualization, Writing—original draft, Writing—review and editing.

**Federica Pecci:** Supervision, Data collection, Visualization, Writing—original draft, Writing—review and editing.

**Xinan Wang:** Supervision, Statistical analysis, Data collection, Visualization, Writing—original draft, Writing—review and editing.

**Maisam Makarem:** Supervision, Data collection, Visualization, Writing—original draft, Writing—review and editing.

**Cassio Murilo Hidalgo Filho:** Supervision, Data collection, Visualization, Writing—original draft, Writing—review and editing.

**Teresa Gorria:** Supervision, Data collection, Visualization, Writing—original draft, Writing—review and editing.

**Arushi Saini:** Data collection, Visualization, Writing—original draft, Writing—review and editing.

**Cindy Pabon:** Supervision, Data collection, Visualization, Writing—original draft, Writing—review and editing.

**James Lindsay:** Supervision, Data collection, Visualization, Writing—original draft, Writing—review and editing.

**Kathleen L. Pfaff:** Supervision, Data collection, Visualization, Writing—original draft, Writing—review and editing.

**Emma L. Welsh:** Supervision, Data collection, Visualization, Writing—original draft, Writing—review and editing.

**Mizuki Nishino:** Supervision, Data collection, Visualization, Writing—original draft, Writing—review and editing.

**Lynette M. Sholl:** Supervision, Data collection, Visualization, Writing—original draft, Writing—review and editing.

**Scott Rodig:** Supervision, Data collection, Visualization, Writing—original draft, Writing—review and editing.

**Saadettin Kilickap:** Supervision, Data collection, Visualization, Writing—original draft, Writing—review and editing.

**Petra Rietschel:** Conceptualization, Methodology, Project administration, Supervision, Visualization, Writing—original draft, Writing—review and editing.

**Debra AG McIntyre:** Supervision, Statistical analysis, Data collection, Visualization, Writing—original draft, Writing—review and editing.

**Jean-Francois Pouliot:** Conceptualization, Methodology, Project administration, Supervision, Visualization, Writing—original draft, Writing—review and editing.

**Mehmet Altan:** Supervision, Data collection, Visualization, Writing—original draft, Writing—review and editing.

**Justin F. Gainor:** Conceptualization, Methodology, Project administration, Supervision, Visualization, Writing—original draft, Writing—review and editing.

**John V. Heymach:** Conceptualization, Methodology, Project administration, Supervision, Visualization, Writing—original draft, Writing—review and editing.

**Adam J. Schoenfeld:** Conceptualization, Methodology, Project administration, Supervision, Visualization, Writing—original draft, Writing—review and editing.

**Mark M. Awad:** Conceptualization, Supervision, Writing—original draft, Writing—review and editing.

## Disclosure

Dr. Ricciuti reports serving on the consulting/advisory board of AstraZeneca, Amgen, and Regeneron; receiving honoraria from Targeted Oncology; and receiving speaker fees from AstraZeneca. Dr. Lin reports receiving consulting from Genentech, C4 Therapeutics, Blueprint Medicines, Nuvalent, Bayer, Elevation Oncology, Novartis, Mirati Therapeutics, Regeneron, Pfizer, Takeda, Ellipses Pharma, Hyku BioSciences, AnHeart Therapeutics, Claim Therapeutics, Turning Point Therapeutics, Bristol-Myers Squibb, and Daiichi Sankyo; receiving institutional research funding from Hengrui Therapeutics, Turning Point Therapeutics, Neon Therapeutics, 10.13039/100019393Relay Therapeutics, 10.13039/100004326Bayer, Elevation Oncology, 10.13039/100004337Roche, Linnaeus Therapeutics, Nuvalent, and 10.13039/100004336Novartis. Dr. Altan reports receiving research funding (to institution) from Genentech, Nektar Therapeutics, Merck, GlaxoSmithKline, Novartis, Jounce Therapeutics, Bristol-Myers Squibb, Eli Lilly, Adaptimmune, Shattuck Lab, and Gilead; serving on the advisory board of GlaxoSmithKline, Shattuck Lab, Bristol-Myers Squibb, AstraZeneca, and Insightec; receiving speaker fees from AstraZeneca, Nektar Therapeutics, and SITC; and having participation of safety review committee for Nanobiotix-MDA Alliance and Hengenix. Dr. Zhang reports receiving grants from 10.13039/100004334Merck; grants and personal fees from Johnson and Johnson and Novartis; and personal fees from Bristol-Myers Squibb, AstraZeneca, GenePlus, Innovent, Varian, Catalyst, and Hengrui, outside the submitted work. Dr. Gainor has served as a compensated consultant or received honoraria from Bristol-Myers Squibb, Genentech/Roche, Takeda, Loxo/Lilly, Blueprint Medicine, Gilead, Moderna, AstraZeneca, Mariana Therapeutics, Mirati, Jounce, Merus Pharmaceuticals, Nuvalent, Pfizer, Novocure, AI Proteins, Novartis, Merck, iTeos, Karyopharm, and Silverback Therapeutics; has received research support from 10.13039/100004336Novartis, Genentech/Roche, and Takeda; has received institutional research support from 10.13039/100002491Bristol-Myers Squibb, Palleon, Tesaro, 10.13039/100019533Moderna, Blueprint, Jounce, 10.13039/100007174Array Biopharma, Merck, Adaptimmune, Novartis, and Alexo; has equity in AI Proteins; and has an immediate family member who is an employee with equity at Ironwood Pharmaceuticals. Dr. Heymach reports serving on the advisory committees of Genentech, Mirati Therapeutics, Eli Lilly & Co., Janssen Pharmaceuticals, Boehringer-Ingelheim Pharmaceuticals, Regeneron, Takeda Pharmaceuticals, BerGenBio, Jazz Pharmaceuticals, Curio Science, Novartis, AstraZeneca Pharmaceuticals, BioAlta, Sanofi, Spectrum Pharmaceuticals, GlaxoSmithKline, EMD Serono, BluePrint Medicine, and Chugai Pharmaceutical; receiving research support from 10.13039/100004325AstraZeneca, Boehringer-Ingelheim, 10.13039/100006399Spectrum, Mirati, Bristol-Myers Squibb, and Takeda; and receiving licensing/royalties from Spectrum. Dr. Pouliot reports being an employee at Regeneron and a Regeneron shareholder. Dr. McIntyre reports being an employee at Regeneron. Dr. Awad reports receiving research funding from Bristol-Myers Squibb, Lilly, 10.13039/100004328Genentech, AstraZeneca, and Elva J. and Clayton L. McLaughlin Fund for Lung Cancer Research; receiving consulting fees from/serving on the advisory board of Merck, Bristol-Myers Squibb, Genentech, AstraZeneca, Nektar, Maverick, Blueprint Medicine, Syndax, AbbVie, Gritstone, ArcherDX, Mirati, NextCure, and EMD Serono. Dr. Rietschel reports being an employee at Regeneron and a Regeneron shareholder with patent issued PCT/US2018/018747. Dr. Nishino reports receiving research funding from Merck, Canon Medical Systems, AstraZeneca, and 10.13039/501100022274Daiichi Sankyo; receiving consulting fees from Daiichi Sankyo and AstraZeneca; and receiving honoraria from Roche. Dr. Sholl reports receiving consulting fee from Genentech, Lilly, and GV20 Therapeutics. The remaining authors have no conflict of interests to disclose.
