# Supplementary material for: Three-Year Overall Survival Outcomes and Correlative Analyses in Patients With NSCLC and High (50%–89%) Versus Very High (≥90%) Programmed Death-Ligand 1 Expression Treated With First-Line Pembrolizumab or Cemiplimab
Source: JTO Clin Res Rep. 2024 Apr 12;5(9):100675. doi: 10.1016/j.jtocrr.2024.100675 (PMC11471150; doi:10.1016/j.jtocrr.2024.100675)
Supplement: Supplementary Tables 1-9 and Figures 1-13 [file mmc1.pdf]

Supplementary Figure 1

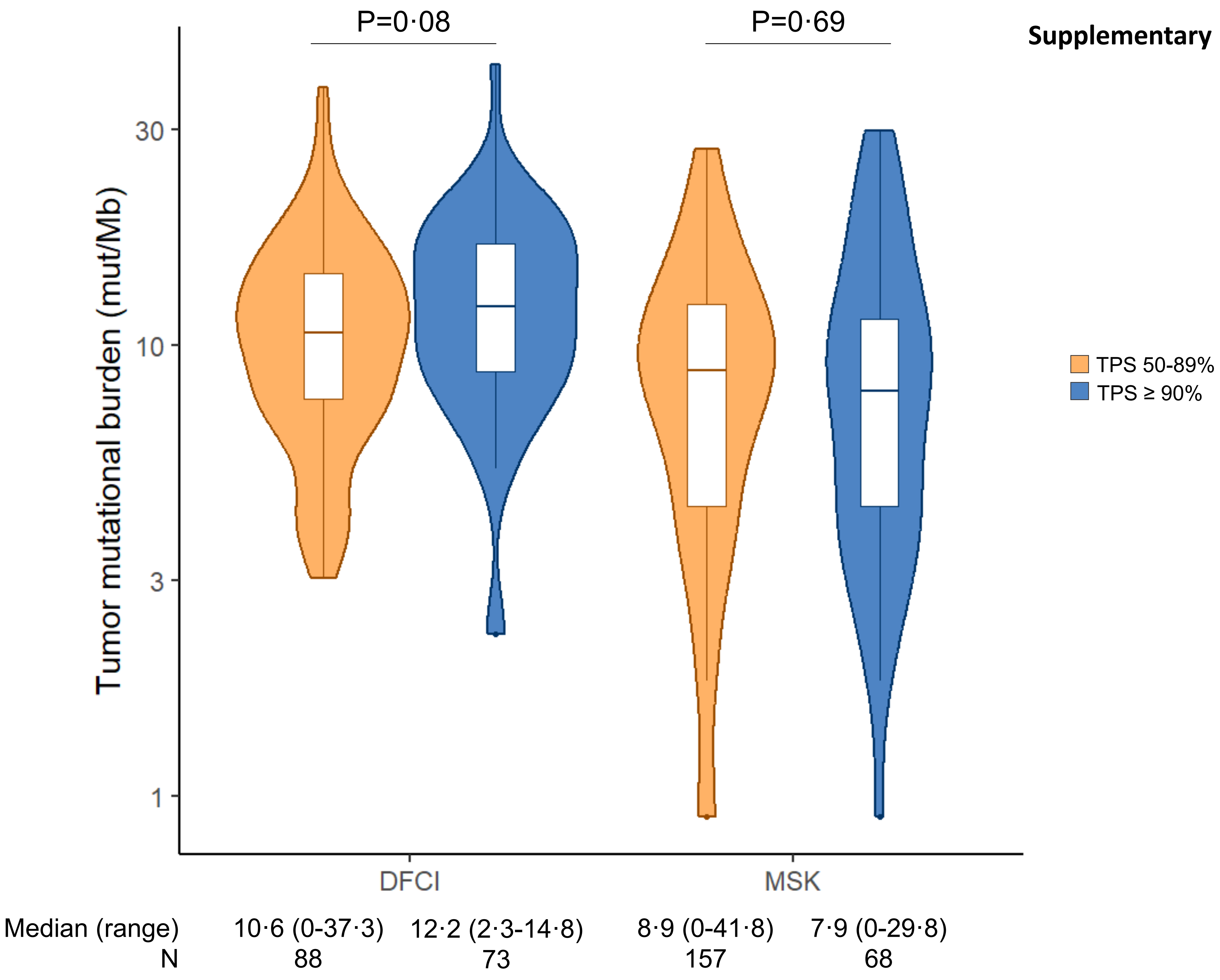

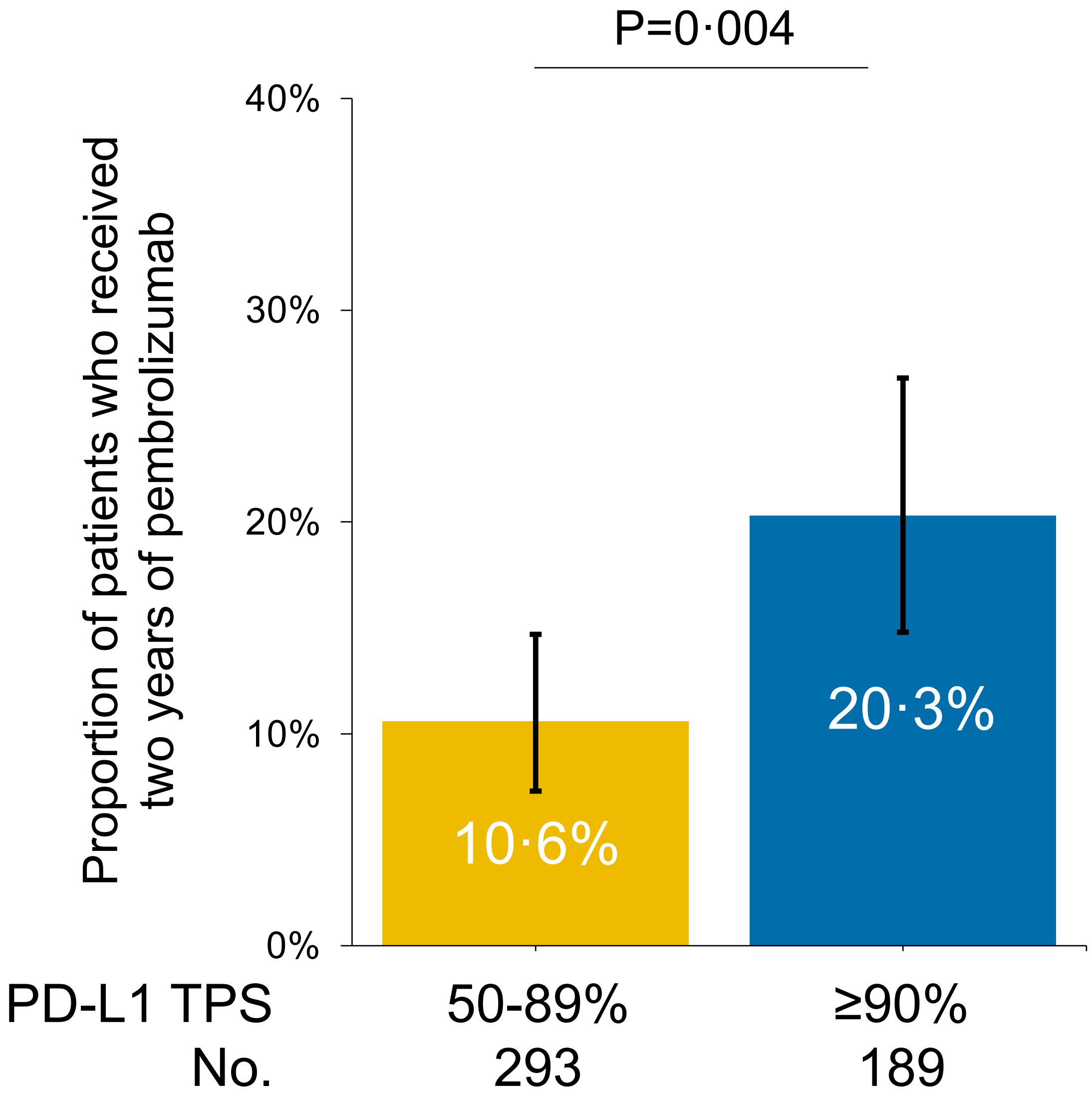

Supplementary Figure 2

A Progression-free survival to pembrolizumab

Subgroup		N	Events	HR	95%CI
Sex	Men	245	192	0.7	0.51 − 0.95
	Women	271	209	0.67	0.51 − 0.89
Age	<70 years	270	200	0.67	0.5 − 0.9
	>70 years	246	201	0.71	0.53 − 0.94
Smoking status	Ever	473	363	0.7	0.56 − 0.87
	Never	43	38	0.8	0.37 − 1.76
ECOG PS	0−1	416	325	0.65	0.52 − 0.82
	>2	100	76	0.87	0.54 − 1.38
Histology	Non−squamous	440	340	0.62	0.49 − 0.77
	Squamous	76	61	1.3	0.76 − 2.23
KRAS	Wild−type	223	175	0.72	0.53 − 0.98
	Mutant	191	147	0.66	0.47 − 0.92
	**Summary**	**516**	**401**	**0.69**	**0.56−0.84**

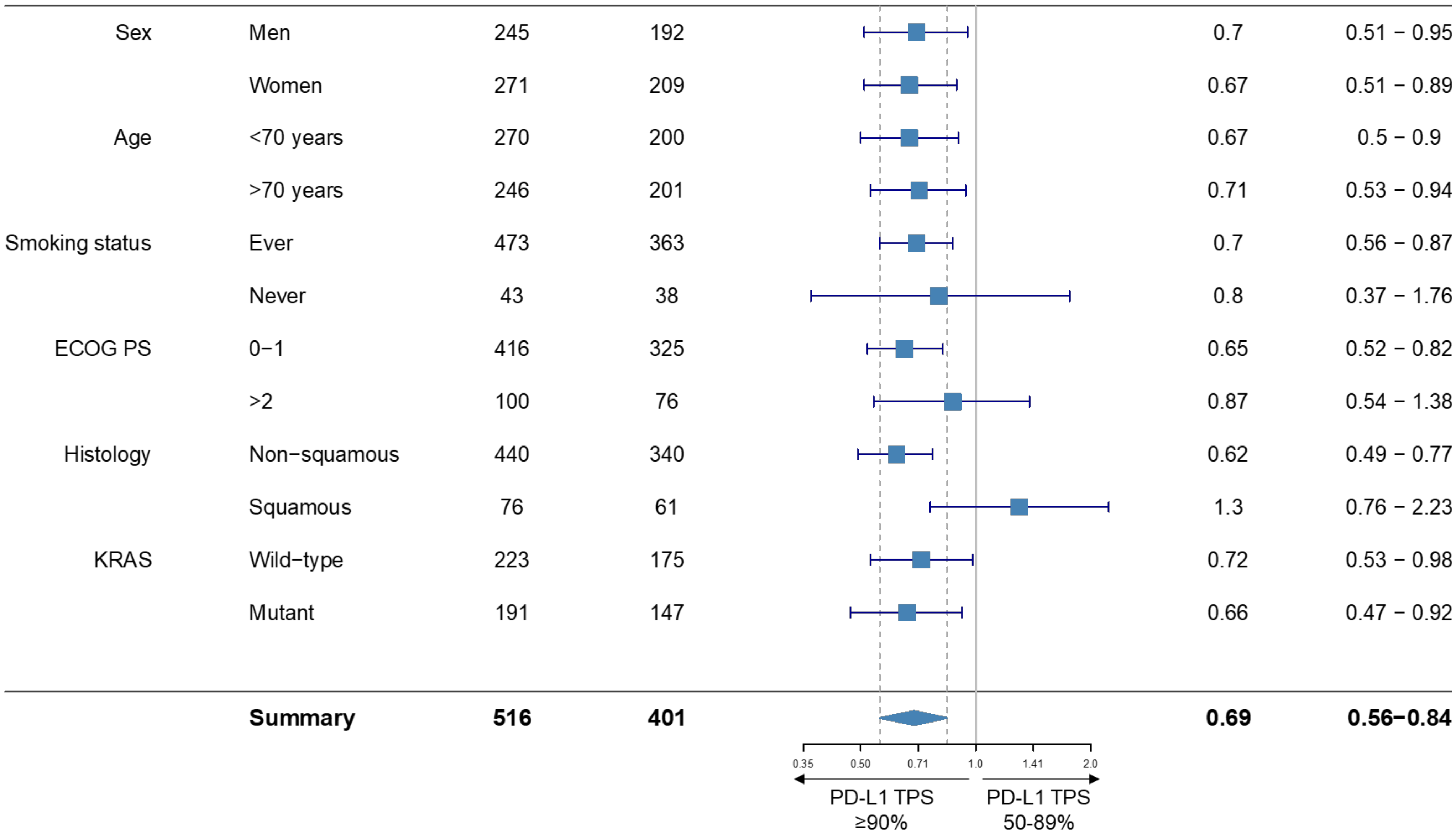

B Overall survival to pembrolizumab

Subgroup		N	Events	HR	95%CI
Sex	Men	245	153	0.62	0.44 − 0.87
	Women	271	167	0.79	0.57 − 1.08
Age	<70 years	270	154	0.73	0.52 − 1.02
	≥70 years	246	166	0.65	0.47 − 0.9
Smoking status	Ever	473	290	0.69	0.54 − 0.88
	Never	43	30	0.95	0.41 − 2.22
ECOG PS	0−1	416	252	0.68	0.52 − 0.88
	≥2	100	68	0.77	0.47 − 1.28
Histology	Non−squamous	440	264	0.64	0.49 − 0.82
	Squamous	76	56	1.39	0.8 − 2.42
KRAS	Wild−type	223	135	0.76	0.53 − 1.08
	Mutant	191	110	0.7	0.48 − 1.04
	**Summary**	**516**	**320**	**0.70**	**0.56−0.88**

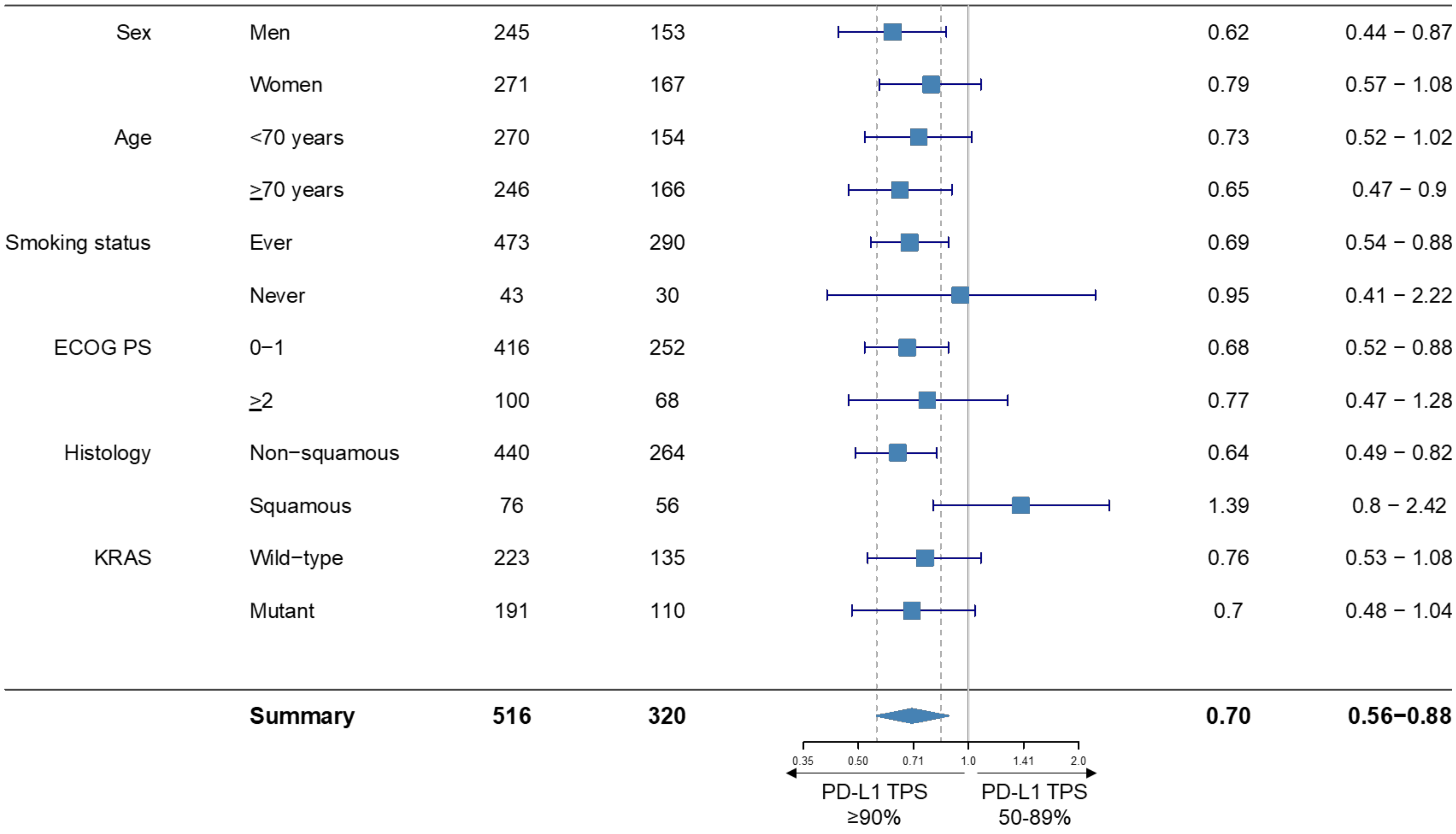

Supplementary Figure 3

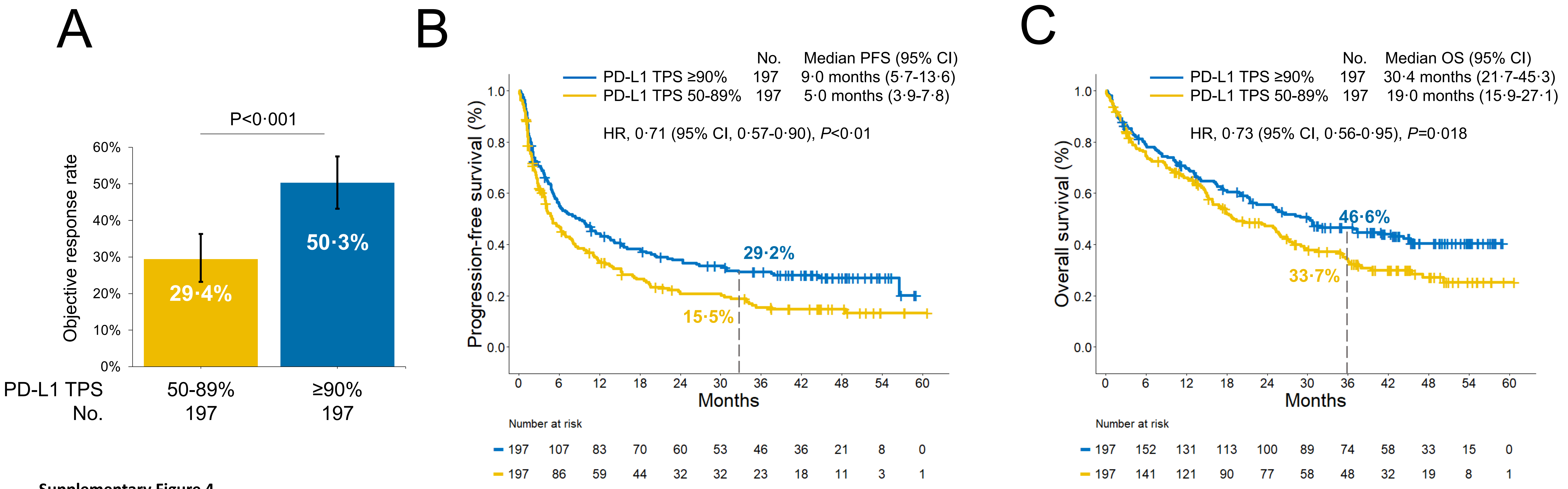

Supplementary Figure 4

Supplementary Figure 5

A Progression-free survival to cemiplimab

Subgroup		N	Events	HR	95%CI
Sex	Men	249	186	0.49	0.36 − 0.68
	Women	35	28	0.95	0.42 − 2.17
Smoking status	Current	106	78	0.47	0.28 − 0.8
	Former	178	136	0.56	0.39 − 0.81
Histology	Squamous	123	97	0.42	0.27 − 0.66
	Non−squamous	161	117	0.61	0.41 − 0.92
ECOG PS	0	77	62	0.35	0.19 − 0.65
	1	207	152	0.61	0.43 − 0.87
	Summary	284	214	0.51	0.38−0.69

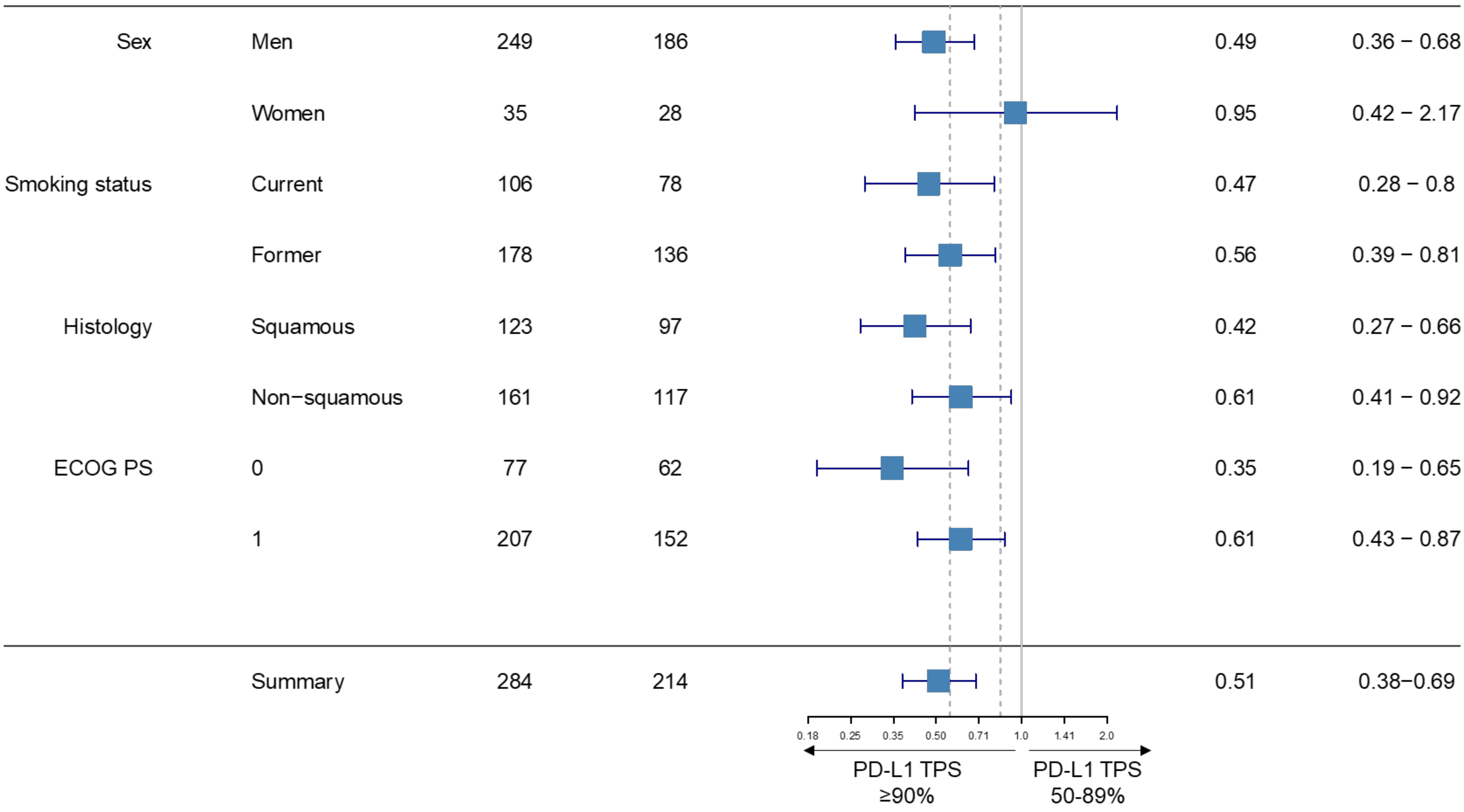

B Overall survival to cemiplimab

Subgroup		N	Events	HR	95%CI
Sex	Men	249	132	0.59	0.4 − 0.87
	Women	35	17	0.9	0.3 − 2.78
Smoking status	Current	106	59	0.73	0.4 − 1.32
	Former	178	90	0.57	0.36 − 0.9
Histology	Squamous	123	71	0.55	0.32 − 0.93
	Non−squamous	161	78	0.67	0.41 − 1.11
ECOG PS	0	77	39	0.27	0.11 − 0.65
	1	207	110	0.79	0.53 − 1.19
	Summary	284	149	0.61	0.42−0.88

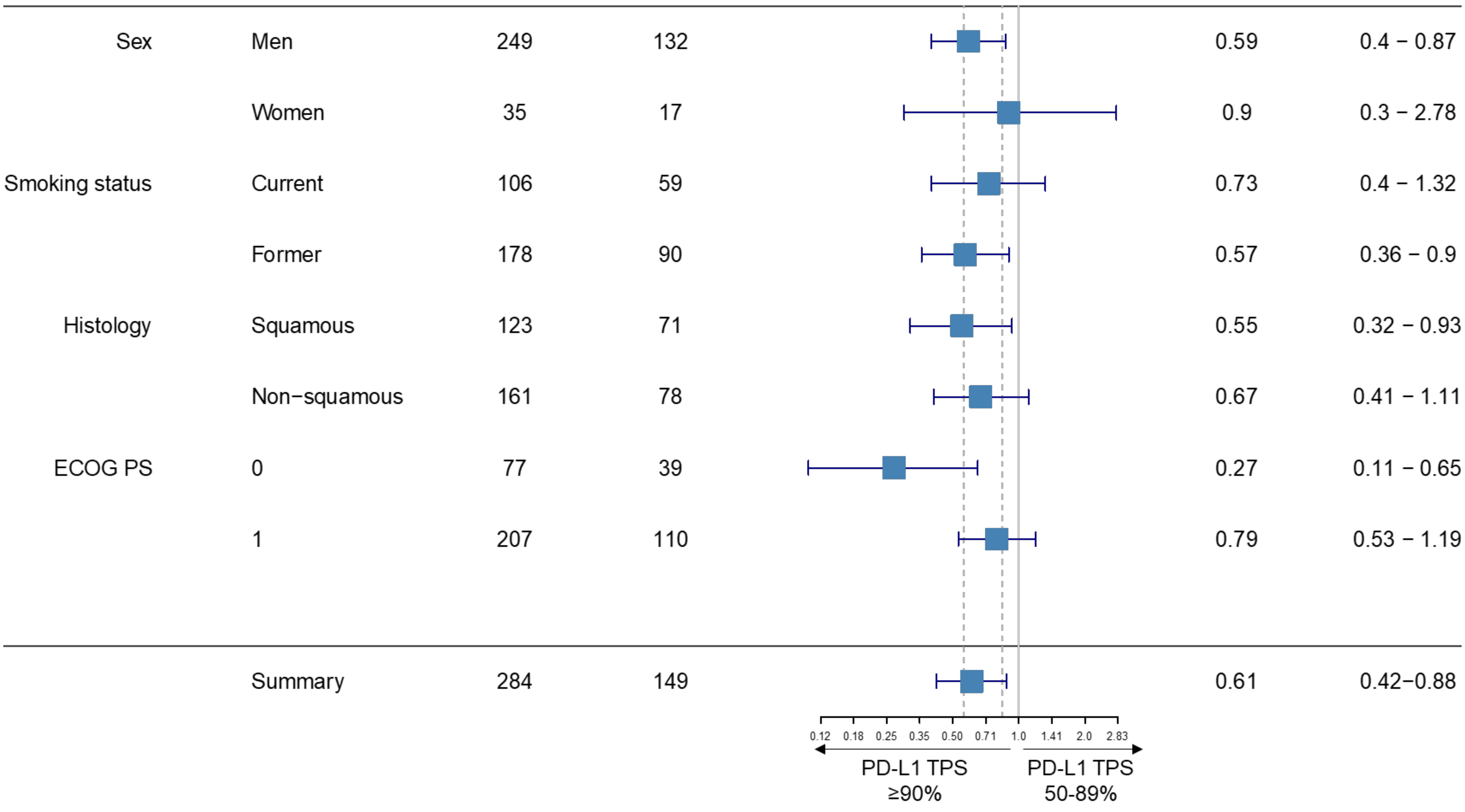

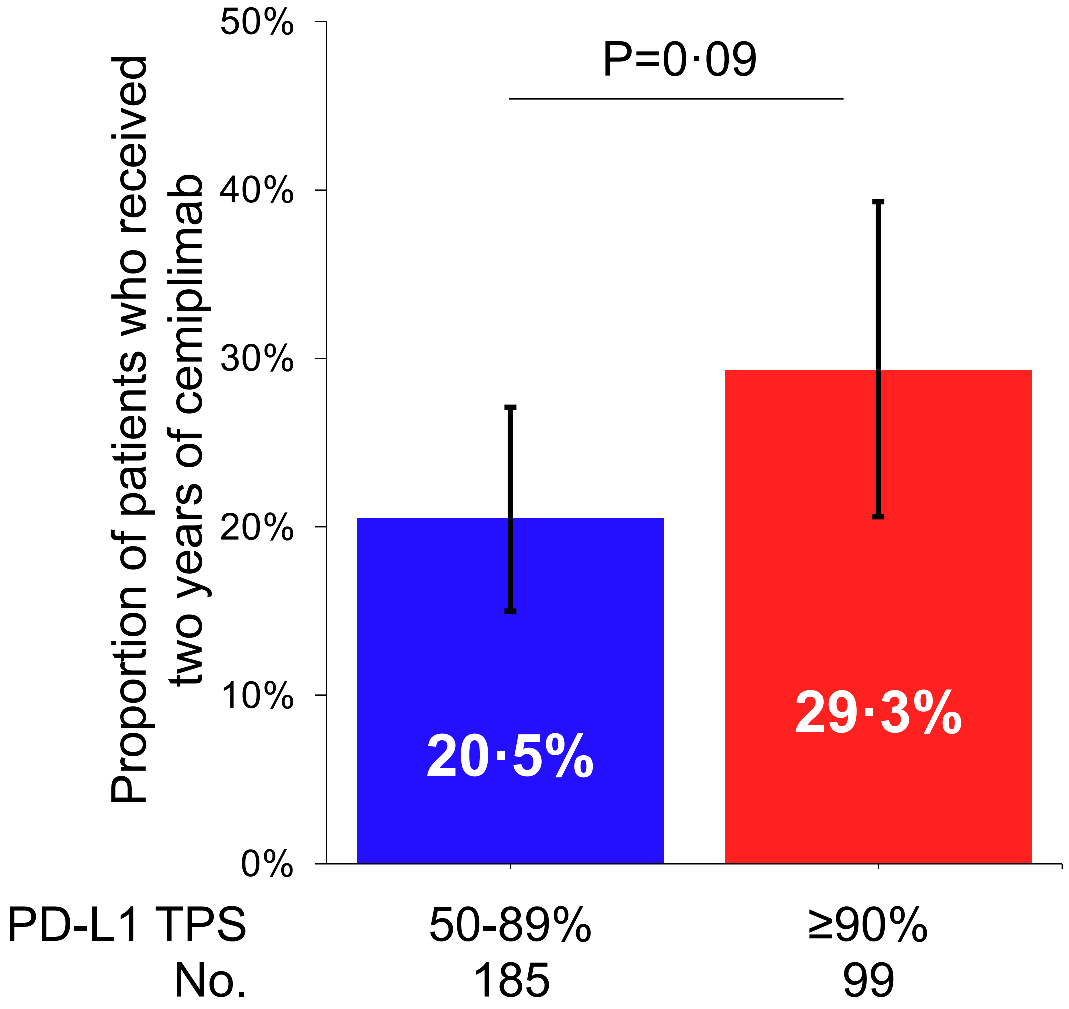

Supplementary Figure 6

A Progression-free survival to chemotherapy

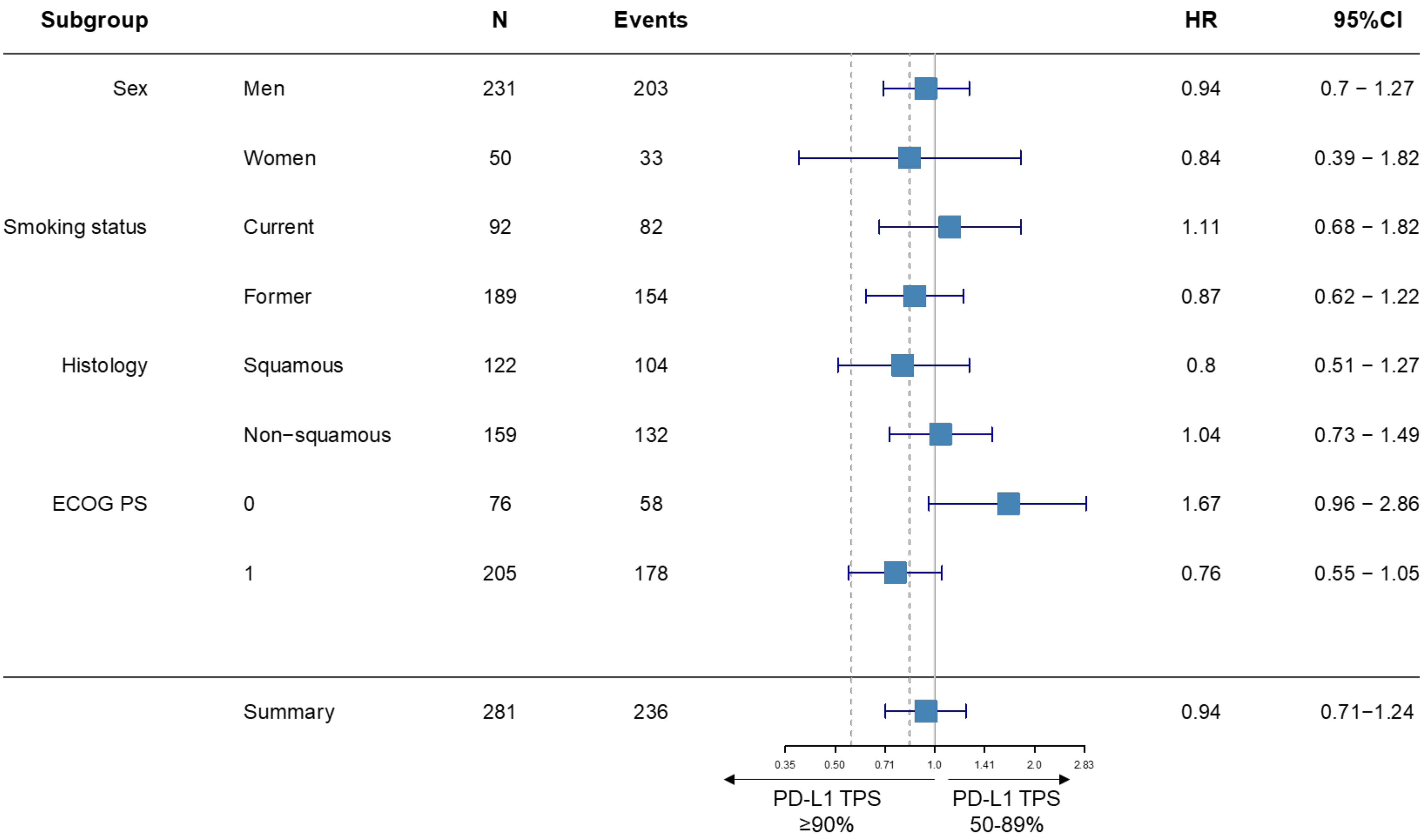

B Overall survival to chemotherapy

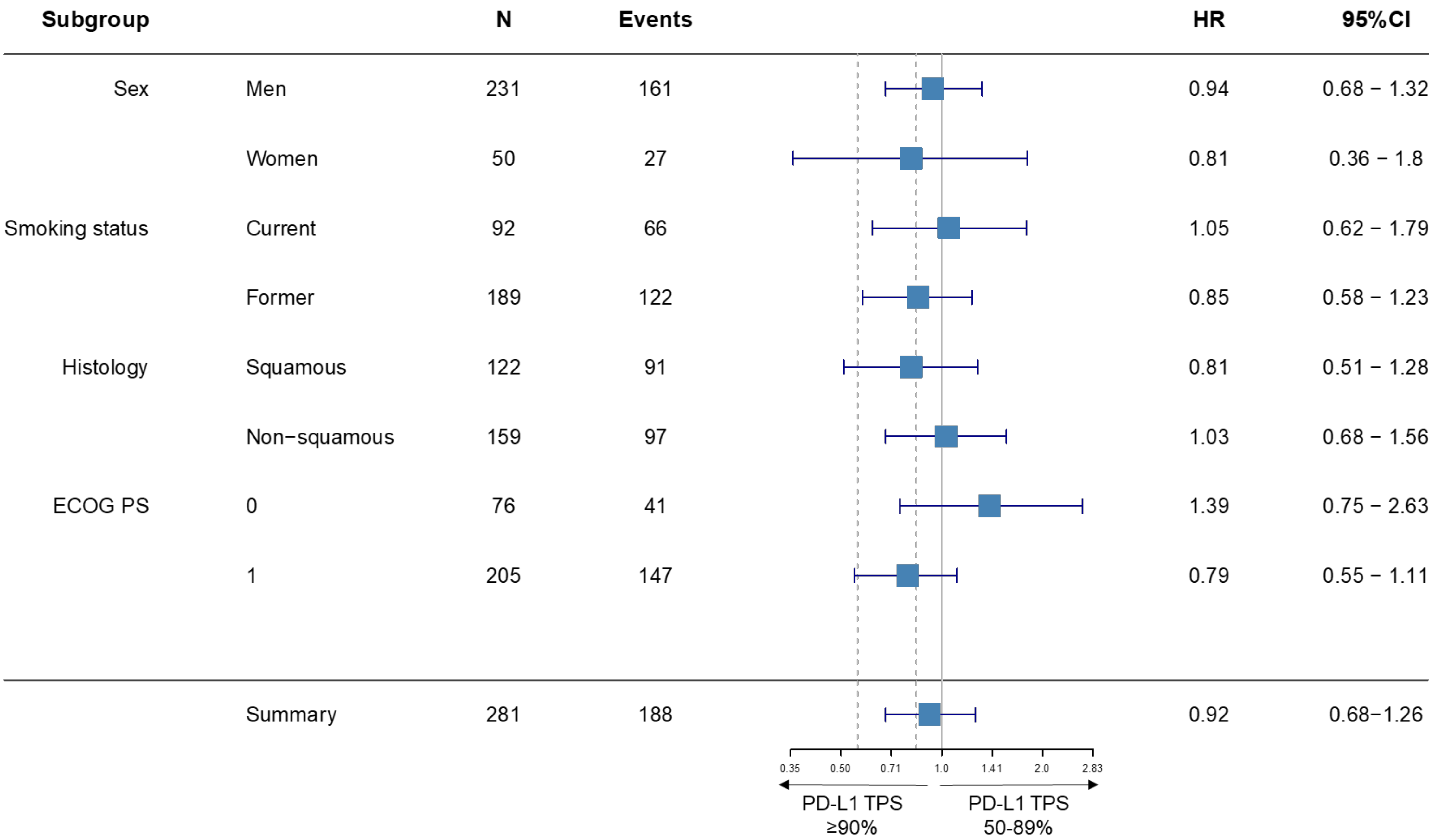

Supplementary Figure 7

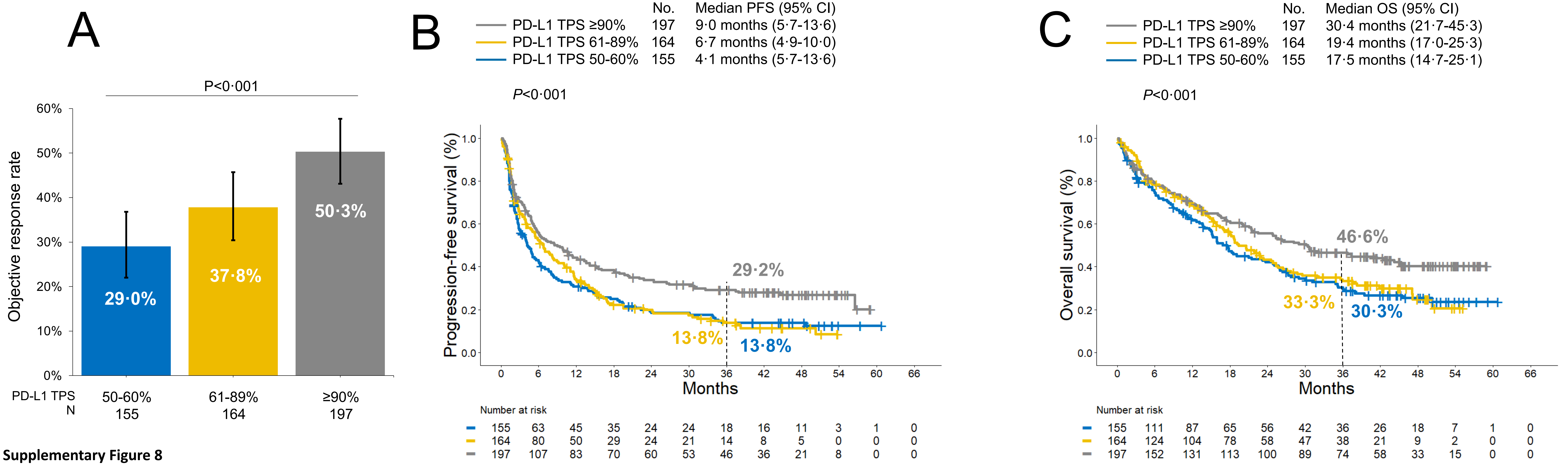

Supplementary Figure 8

Supplementary Figure 9

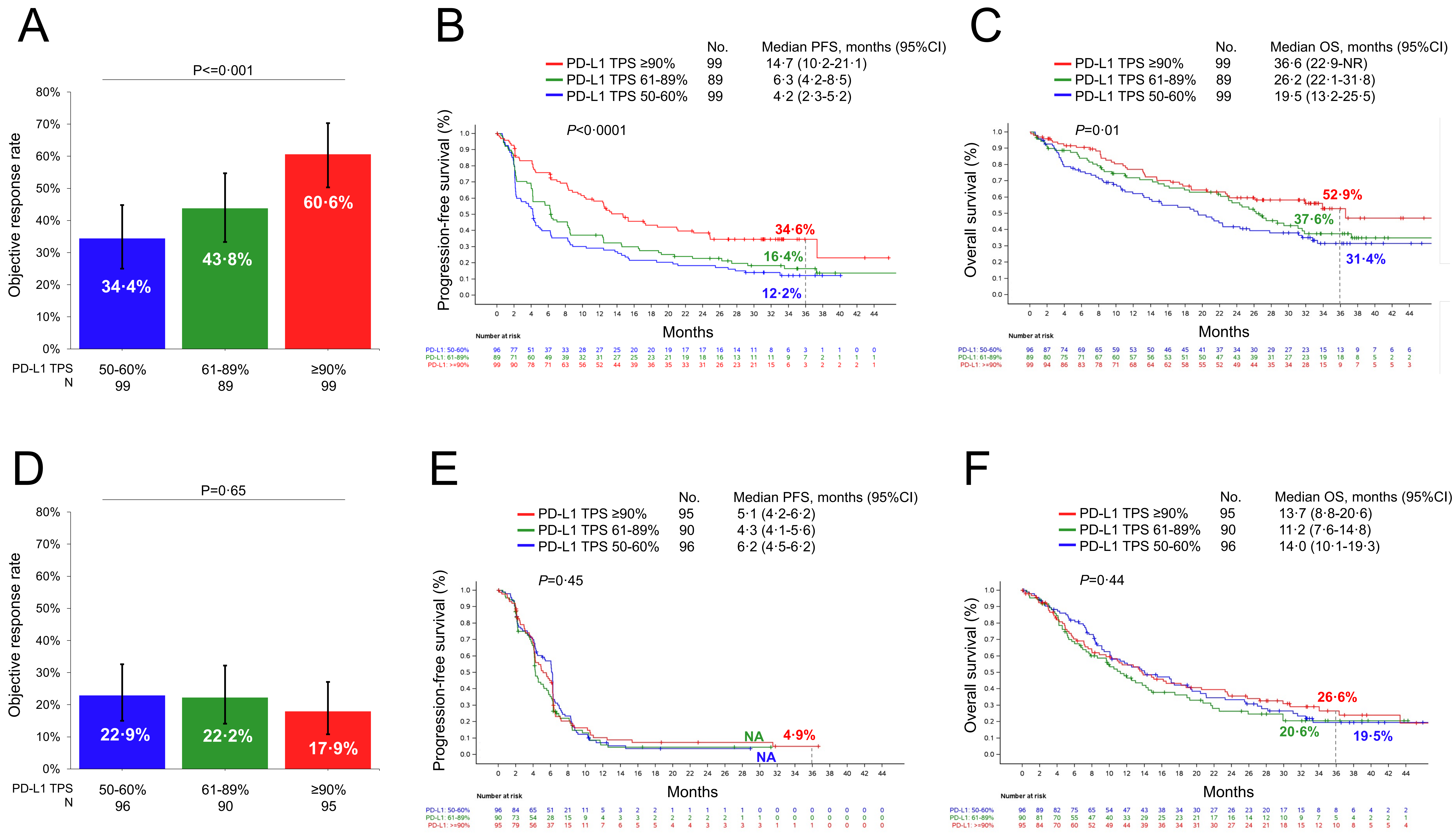

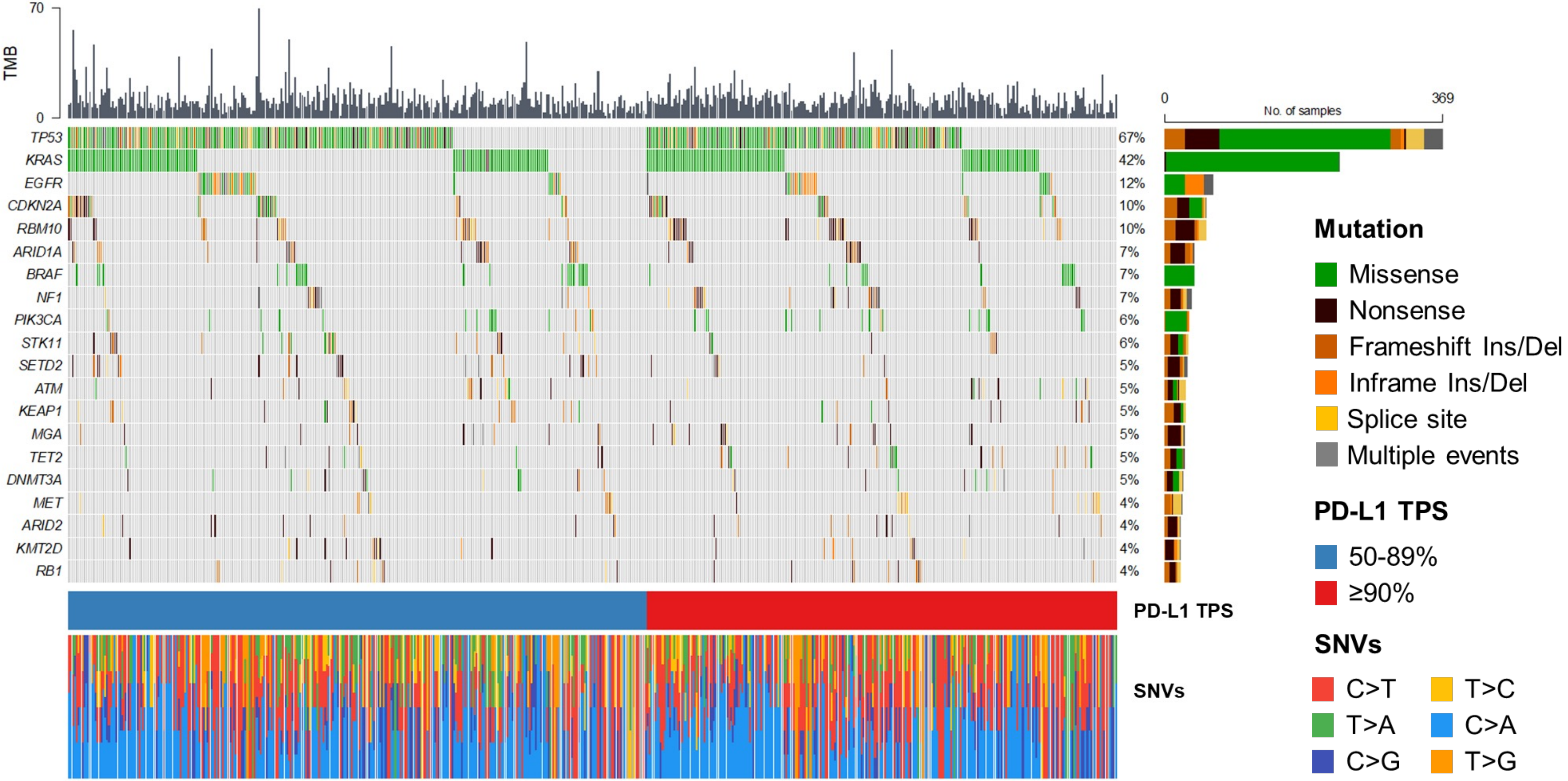

Supplementary Figure 10

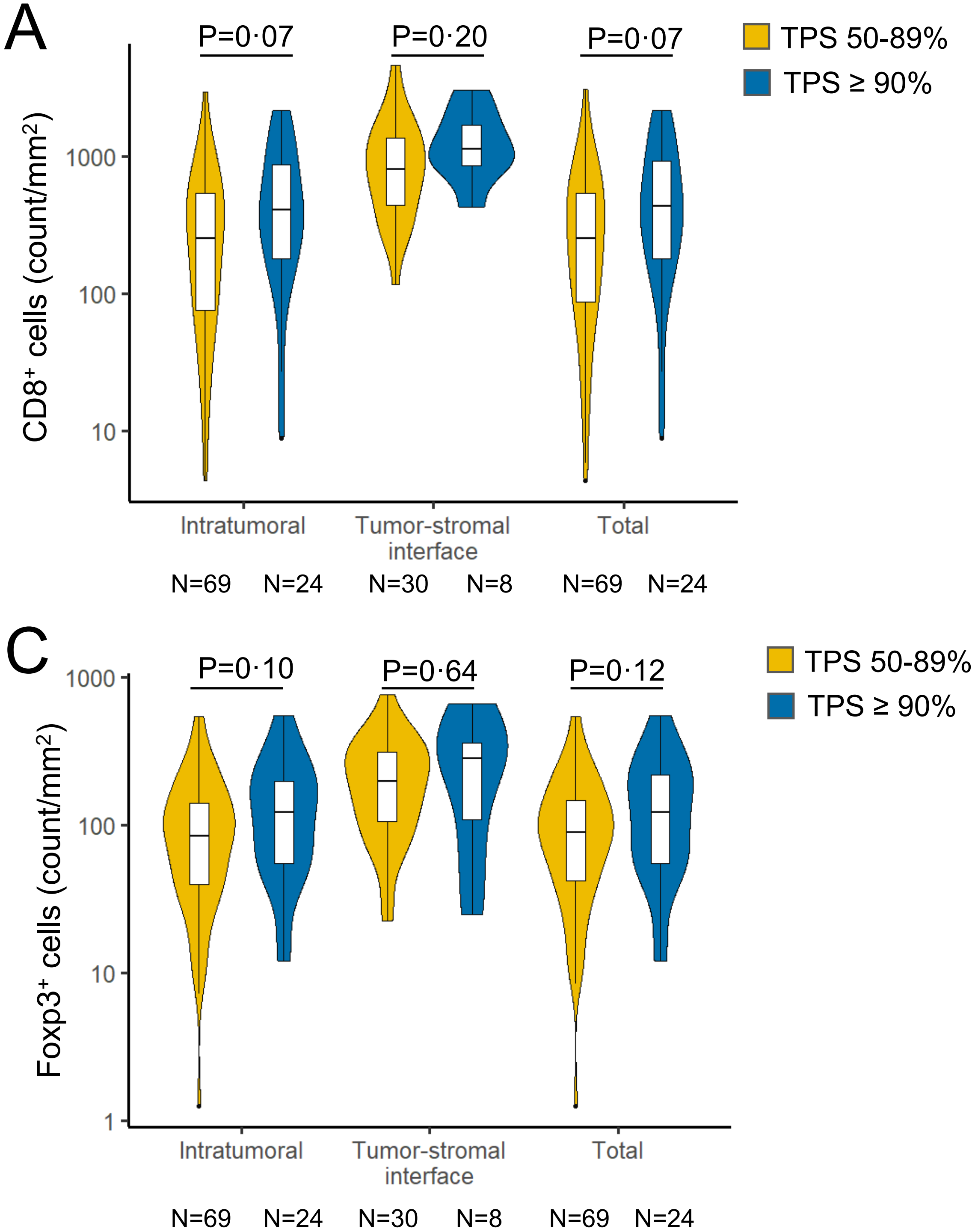

Supplementary Figure 11

Supplementary Figure 12

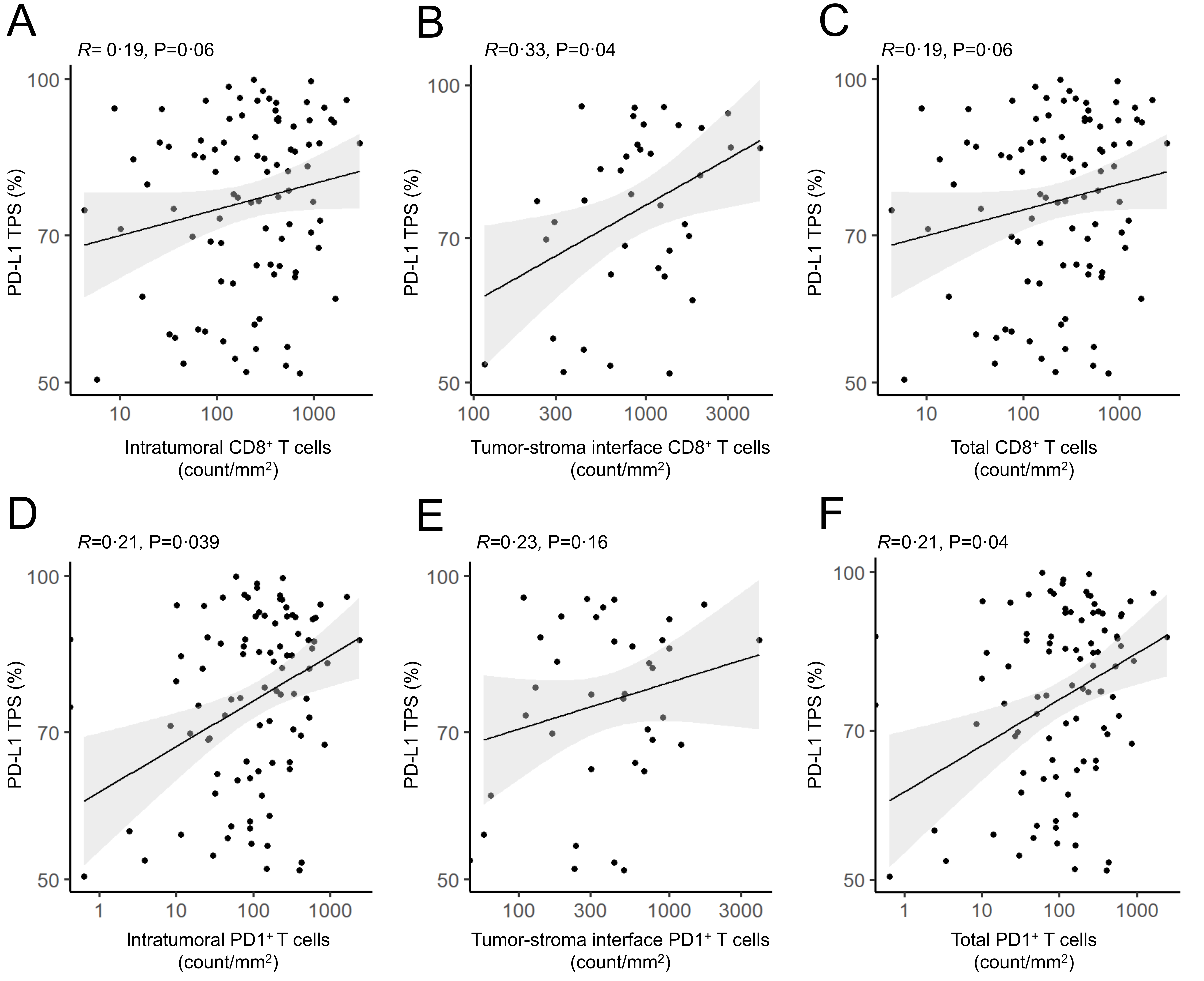

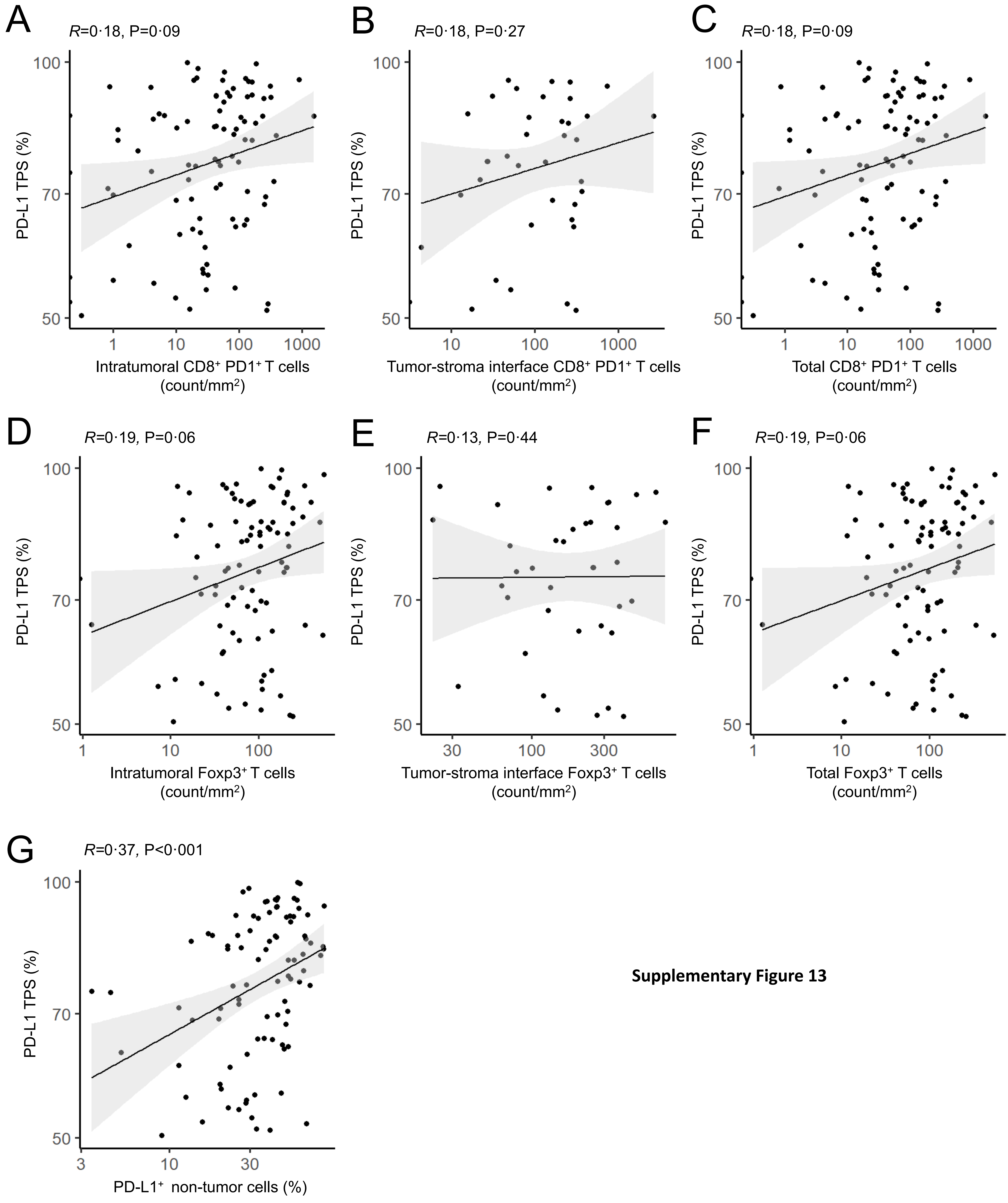

Supplementary Figure 13

Supplementary Table 1. Characteristics of 516 patients with advanced NSCLC who received first-line pembrolizumab in the retrospective, multi-center academic cohort.

Characteristic	N=516 (%)
**Age**, median (range)	69 (30-93)
**Sex**	
• Men	245 (47·5)
• Women	271 (52·5)
**Smoking history**	
• Ever	473 (91·7)
• Never	43 (8·3)
**Pack/years**, median (range)	30 (0-208)
**Histology**	
• Non-squamous	440 (85·3)
• Squamous	76 (14·7)
**ECOG PS**	
• 0-1	416 (80·6)
• ≥2	100 (19·4)
**Driver**	
• KRAS	191 (46·1)
• BRAF	21 (5·1)
• MET	14 (3·4)
• RET	1 (0·2)
• None identified	187 (45·2)
• Not assessed	102
**PD-L1 TPS**	
• 50-89%	319 (61·8)
• ≥90%	197 (38·2)
**Institution**	
• MSKCC	225 (43·6)
• DFCI	161 (31·2)
• MGH	67 (13·0)
• MDACC	63 (12·2)

ECOG PS, Eastern Cooperative Oncology Group Performance Status

Supplementary Table 2. Characteristics of 565 patients with advanced NSCLC who received first-line cemiplimab or chemotherapy in the prospective EMPOWER-Lung-01 phase III, randomized clinical trial.

**Characteristic**	**Cemiplimab arm N=284 (%)**	**Chemotherapy arm N=281 (%)**
**Age**, median (range)	63 (31-79)	64 (40-84)
**Sex**		
• Men	249 (87·7)	231 (82·2)
• Women	35 (12·3)	50 (17·8)
**Histology**		
• Non-squamous	161 (56·7)	159 (56·6)
• Squamous	123 (43·3)	122 (43·4)
**ECOG PS**		
• 0	77 (27·1)	76 (27·0)
• 1	207 (72·9)	205 (73·0)
**Region of enrollment**		
• Europe	216 (76·1)	217 (77·2)
• Asia	31 (10·9)	29 (10·3)
• Rest of the world	37 (13·0)	35 (12·5)
**Smoking status**		
• Current	106 (37·3)	92 (32·7)
• Former	178 (62·7)	189 (67·3)
**Cancer stage at screening**		
• IIIB/C	45 (15·8)	42 (15·0)
• IV	239 (84·2)	239 (85·0)
**PD-L1 TPS**		
• 50-89%	185 (65·1)	186 (66·2)
• ≥90%	99 (34·9)	95 (33·8)

ECOG PS, Eastern Cooperative Oncology Group Performance Status

Supplementary Table 3. Characteristics of patients with advanced NSCLC who received first-line pembrolizumab according to PD-L1 expression levels (50-89% versus ≥90%).

**Characteristics**	**PD-L1 TPS 50-89% N=319**	**PD-L1 TPS ≥90% N=197**	**P**
**Age**, median (range)	69 (35-93)	69 (30-88)	0·71
**Sex**			
• Male	158 (49·5)	87 (44·2)	0·24
• Female	161 (50·5)	110 (55·8)	
**Smoking history**			
• Ever	286 (89·7)	187 (94·9)	0·047
• Never	33 (10·3)	10 (5·1)	
**Pack/years**, median (range)	33·8 (0-200)	30 (0-208)	0·87
**Histology**			
• Non-squamous	266 (83·4)	174 (88·3)	0·16
• Squamous	53 (16·6)	23 (11·7)	
**ECOG PS**			
• 0-1	257 (80·6)	159 (80·7)	0·99
• ≥2	62 (19·4)	38 (19·3)	
**Driver**			
• KRAS	110 (44·5)	81 (48·5)	0·34
• BRAF	14 (5·7)	7 (4·2)	
• MET	6 (2·4)	8 (4·8)	
• RET	0 (0·0)	1 (0·6)	
• None identified	117 (47·4)	70 (41·9)	
• Not assessed	72	30	

PS ECOG, Performance Status Eastern Cooperative Oncology Group

Supplementary Table 4. Multivariable Cox regression for progression-free and overall survival in the first-line pembrolizumab cohort.

	**Progression-free survival**	**Overall survival**
**Variable**	Multivariable HR [95% CI], P	Multivariable HR [95% CI], P
Age*	1·00 [0·99-1·01], 0·66	1·01 [1·00-1·02], 0·002
Sex (male versus female)	1·00 [0·81-1·23], 0·98	0·98 [0·78-1·24], 0·89
ECOG PS (≥2 versus 0-1)	1·37 [1·07-1·77], 0·01	1·78 [1·36-2·33], <0·0001
Histology (squamous versus non-squamous)	0·99 [0·75-1·32], 0·99	1·29 [0·95-1·75], 0·09
Smoking history (never versus ever)	1·75 [1·24-2·46], 0·001	1·32 [0·90-1·95], 0·14
PD-L1 TPS (≥90% versus 50-89%)	0·70 [0·57-0·87], 0·001	0·73 [0·58-0·93], 0·01

ECOG PS, Eastern Cooperative Oncology Group Performance Status

*Age is included in the regression model as a continuous variable

Supplementary Table 5. Multivariable Cox regression for progression-free and overall survival to pembrolizumab in the propensity-score matched cohort.

	**Progression-free survival**	**Overall survival**
**Variable**	Multivariable HR [95% CI], P	Multivariable HR [95% CI], P
Age*	1·00 [0·99-1·01], 0·32	1·01 [1·00-1·03], 0·007
Sex (male versus female)	1·02 [0·80-1·29]. 0·86	0·95 [0·73-1·25], 0·75
ECOG PS (≥2 versus 0-1)	1·44 [1·08-1·90], 0·01	1·76 [1·30-2·39], <0·001
Histology (squamous versus non-squamous)	1·05 [0·72-1·53], 0·79	1·38 [0·92-2·05], 0·11
Smoking history (never versus ever)	1·99 [1·21-3·29], 0·006	1·44 [0·83-2·50], 0·19
PD-L1 TPS (≥90% versus 50-89%)	0·72 [0·58-0·91], 0·007	0·75 [0·58-0·97], 0·03

ECOG PS, Eastern Cooperative Oncology Group Performance Status

*Age is included in the regression model as a continuous variable

Supplementary Table 6. Characteristics of 284 patients with advanced NSCLC who received first-line cemiplimab in the EMPOWER-Lung 1 trial by PD-L1 expression levels.

**Characteristic***	**PD-L1 TPS 50-89% N=185 (%)**	**PD-L1 TPS ≥90% N=99 (%)**	**P**
**Age**, median (range)	63 (31-79)	64 (43-79)	0·55
**Sex**			
• Male	160 (86·5)	89 (89·9)	0·45
• Female	25 (13·5)	10 (10·1)	
**Histology**			
• Non-squamous	105 (56·8)	56 (56·6)	0·95
• Squamous	80 (43·2)	43 (43·4)	
**ECOG PS**			
• 0	52 (28·1)	25 (25·2)	0·68
• 1	133 (71·9)	74 (74·7)	
**Smoking status**			
• Current	73 (39·5)	33 (33·3)	0·37
• Former	112 (60·5)	66 (66·7)	

*Driver mutation status was not available for this cohort.

ECOG PS, Eastern Cooperative Oncology Group Performance Status

Supplementary Table 7. Characteristics of 281 patients with advanced NSCLC who received first-line platinum-based chemotherapy in the EMPOWER-Lung 1 phase III, randomized clinical trial by PD-L1 expression levels.

**Characteristic***	**PD-L1 TPS 50-89% N=186 (%)**	**PD-L1 TPS ≥90% N=95 (%)**	**P**
**Age**, median (range)	64 (40-81)	35 (46-84)	0·26
**Sex**			
• Male	153 (82·3)	78 (82·1)	0·95
• Female	33 (17·7)	17 (17·9)	
**Histology**			
• Non-squamous	102 (54·8)	57 (60·0)	0·45
• Squamous	84 (45·2)	38 (40·0)	
**ECOG PS**			
• 0	48 (25·8)	28 (29·5)	0·57
• 1	138 (74·2)	67 (70·5)	
**Smoking status**			
• Current	65 (34·9)	27 (28·4)	0·29
• Former	121 (65·1)	68 (71·6)	

*Driver mutation status was not available for this cohort.

ECOG PS, Eastern Cooperative Oncology Group Performance Status

Supplementary Table 8. Baseline clinicopathologic characteristics of patients with non-small cell lung cancer with PD-L1 TPS 50-89% versus those with PD-L1 TPS ≥90% whose tumors underwent comprehensive tumor genomic profiling at DFCI.

Clinical Characteristic, n (%)	PD-L1 TPS 50-89% N=305	PD-L1 TPS ≥90% N=248	P-value
**Age, median (range)**	67 (29-98)	67 (27-90)	0·90
**Sex**			0·79
• Male	118 (38·7%)	99 (39·9%)	
• Female	187 (61·3%)	149 (60·1%)	
**Smoking status**			0·03
• Current/Former	243 (79·7%)	215 (86·7%)	
• Never	62 (20·3%)	33 (13·3%)	
**Pack/Years, median (range)**	23 (0-200)	25 (0-120)	0·16
**Histology**			1·0
• Non-squamous	283 (92·8%)	230 (92·7%)	
• Squamous	22 (7·2%)	18 (7·6%)	
**TMB, mut/Mb**	9·9 (0-70·2)	10·9 (1·5-43·3)	0·22
**Stage at time of sequencing**			0·96
• I	19 (6·23%)	16 (6·45%)	
• II	11 (3·61%)	7 (2·82%)	
• III	32 (10·5%)	26 (10·5%)	
• IV	243 (79·7%)	199 (80·2%)	

Supplementary Table 9. Baseline clinicopathologic characteristics of patients with non-small cell lung cancer with PD-L1 TPS 50-89% vs ≥90% whose tumors underwent multiplexed immunofluorescence at DFCI.

Clinical Characteristic, n (%)	PD-L1 TPS 50-89% N=69	PD-L1 TPS ≥90% N=24	P-value
**Age, median (range)**	68 (29-85)	71 (28-85)	0·33
**Sex**			
• Male	44 (63·8%)	14 (58·3%)	0·63
• Female	25 (36·2%)	10 (41·7%)	
**Smoking status**			
• Current/Former	57 (82·6%)	21 (87·5%)	1
• Never	11 (15·9%)	3 (12·5%)	
• Not available	1		
**Pack-years, median (range)**	32·5 (3-90)	40 (15-122)	0·31
**Histology**			
• Nonsquamous	58 (84·1%)	19 (79·2%)	0·55
• Squamous	11 (15·9%)	5 (20·8%)	
**Stage at ImmunoProfile**			
• I	17 (24·6%)	3 (12·5%)	
• II	8 (11·6%)	3 (12·5%)	0·43
• III	10 (14·5%)	2 (8·3%)	
• IV	34 (49·3%)	16 (66·7%)	
**Site of biopsy**			
• Primary lung tissue	46 (66·7%)	15 (62·5%)	0·80
• Metastatic tissue	23 (33·3%)	9 (37·5%)	
